# Effects of model inaccuracies on reaching movements with intermittent control

**DOI:** 10.1371/journal.pone.0224265

**Published:** 2019-10-30

**Authors:** Igor Gindin, Miri Benyamini, Miriam Zacksenhouse

**Affiliations:** Faculty of Mechanical Engineering, Technion Israel’s Institute of Technology, Haifa 32000, Israel; Universita degli Studi di Napoli Federico II, ITALY

## Abstract

**Background and objectives:**

Human motor control (HMC) has been hypothesized to involve state estimation, prediction and feedback control to overcome noise, delays and disturbances. However, the nature of communication between these processes, and, in particular, whether it is continuous or intermittent, is still an open issue. Depending on the nature of communication, the resulting control is referred to as continuous control (CC) or intermittent control (IC). While standard HMC theories are based on CC, IC has been argued to be more viable since it reduces computational and communication burden and agrees better with some experimental results. However, to be a feasible model for HMC, IC has to cope well with inaccurately modeled plants, which are common in daily life, as when lifting lighter than expected loads. While IC may involve event-driven triggering, it is generally assumed that refractory mechanisms in HMC set a lower limit on the interval between triggers. Hence, we focus on periodic IC, which addresses this lower limit and also facilitates analysis.

**Theoretical methods and results:**

Theoretical stability criteria are derived for CC and IC of inaccurately modeled linear time-invariant systems with and without delays. Considering a simple muscle-actuated hand model with inaccurately modeled load, both CC and IC remain stable over most of the investigated range, and may become unstable only when the actual load is much smaller than expected, usually smaller than the minimum set by the actual mass of the forearm and hand. Neither CC nor IC is consistently superior to the other in terms of the range of loads over which the system remains stable.

**Numerical methods and results:**

Numerical simulations of time-delayed reaching movements are presented and analyzed to evaluate the effects of model inaccuracies when the control and observer gains are time-dependent, as is assumed to occur in HMC. Both IC and CC agree qualitatively with previously published experimental results with inaccurately modeled plants. Thus, our study suggests that IC copes well with inaccurately modeled plants and is indeed a viable model for HMC.

## Introduction

Stochastic optimal feedback control (OFC) has been strongly advocated as a framework for investigating human motor control (HMC) [1–5]. The inherent noise in HMC is handled by an optimal observer that estimates the state, while inherent delays are overcome by a predictor that predicts the current state from time-delayed state estimation [3, 6]. Standard HMC theories assume that the communication between these processes is continuous, though others argue that it is intermittent [5, 7–10]. Depending on whether the communication is continuous or intermitent, the resulting control is referred to as continuous control (CC) or intermittent control (IC), respectively. IC is advantageous since it reduces the computational and communication load [5, 11]. In the control literature, IC is known as sampled data systems (SDC) [11, 12], and is regaining interest for decentralized control. In the context of HMC, IC was suggested more than half a century ago to explain the psychological refractory period in tracking hand movements [13, 14]. However, the robustness of IC to model inaccuracies has not been addressed.

**Fig 1:**
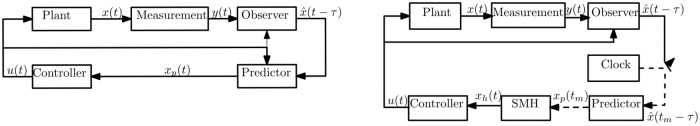
Block diagram of continuous control (CC, left) and periodic intermittent control (periodic IC, right) with system-matched hold (SMH) [5]. Solid and dashed lines carry continuous and intermittent communication, respectively. Due to measurement delay *τ*, the current measurement, *y*(*t*), reflects the state of the plant, *x*, at *t* − *τ*, so the observer can only estimate the observer state $\hat{x}\left(t-\tau \right)$. In CC, the predictor operates continuousely to generate the predicted state *x*_*p*_(*t*) from which the control signal *u*(*t*) is derived. In periodic IC, the predictor operates only at the evenly spaced triggers generated by the clock. At the time of the *m*–th trigger, *t*_*m*_, the predictor generates *x*_*p*_(*t*_*m*_) that provides the initial condition to the SMH. The SMH generates the hold state *x*_*h*_(*t*), which approximates the predicted state in open-loop based on the dynamics of the closed-loop system. External inputs, reflecting task goal, noise and disturbances, are omitted for clarity.

CC involves three main processes, as depicted in the left panel of Fig 1 (and further detailed in Continuous control): (i) *Observer* that combines the current noisy and delayed sensory measurement, *y*(*t*), with an internal model to estimate the time-delayed state $\hat{x}\left(t-\tau \right)$, where *τ* is the delay; (ii) *Predictor* that generates the predicted state, *x*_*p*_(*t*), given $\hat{x}\left(t-\tau \right)$; and (iii) *Controller* that derives the control signal *u*(*t*) from *x*_*p*_(*t*). IC requires two additional processes, as depicted in the right panel of Fig 1 (and further detailed in Intermittent control) [5, 12, 15]: (iv) *Trigger* that initiates information transfer between the observer and predictor at discrete times, *t*_*m*_ (*m* = 1, 2, …), which are evenly spaced (clock-driven, periodic IC) or determined by specific events (event-driven, not considered here); and (v) *System-Matched Hold (SMH)* that generates the hold state *x*_*h*_(*t*), which approximates the predicted state in open-loop based on the internal model and the known control law. SMH facilitates IC since it accounts for the known control law between samples, rather than assuming that the control signal between samples is constant, as in the more common zero-order hold (ZOH).

Triggers are assumed to have a range of roles in HMC, including movement initiation (via the Go signal, for example) and transitions from one phase of locomotion to another [16, 17]. However, the main aspect of IC considered here is the use of SMH to approximate the predicted state in open-loop, in order to reduce the computational and communication burden. IC can explain important observations including: (i) highly variable timing of corrective sub-movements [18]; (ii) response to double stimuli with narrow pulses [5, 19], (iii) multi-peaked velocity profiles even in the presence of Gaussian noise [20] (though this can also be explained by CC with non-Gaussian process noise); and (iv) bursting activity in neural recording [21].

We focus on periodic IC, which involves clock-driven triggering. While HMC may involve event-driven triggering [14, 15, 22], it is generally assumed that refractory mechanisms set a lower limit on the sampling period [5, 13]. Hence, our analysis pertains to periodic IC at that lower limit.

To be a viable candidate for HMC, IC has to cope with inaccurately modeled plants, i.e., plants that differ from their internal model. Inaccurate models are common in daily activities, as when lifting lighter than expected loads or attempting to turn a locked steering wheel. Inaccurate models may also occur when cerebellar patients fail to update their internal models [23]. Thus, our main objective was to evaluate and compare the effects of plant inaccuracies on IC and CC, and thus assess whether IC is a viable model for HMC. This was accomplished using both theoretical analysis and numerical simulations. Theoretical analysis was facilitated by considering time-invariant systems, which result from optimal control of time-invariant plants with respect to cost functions with time-invariant cost matrices. Numerical simulations were used to investigate movements with time-varying controller and observer gains, which are optimal when the cost function involve changing cost matrices, and in particular, when the cost matrices change at the desired reaching time. In either case, the controller and observer gains were determined using standard optimal control and estimation tools, as if the plant is accurately modeled.

Our second objective was to evaluate whether OFC, with either CC or IC, can provide an alternative model for the experimental results reported by Bhanpuri *et al*. [23], which involve reaching movements with inaccurately modeled plants. We demonstrate that both CC and IC reproduce well the main experimental results in [23], and in particular the observed overshoot and undershoot with heavier and lighter than expected mass, respectively.

## Results

Results include (i) theoretical criteria for determining the stability of CC or IC of inaccurately modeled linear time-invariant (LTI) plants with and without delays, under the assumption that the feedback and observer gains are also time-invariant, (ii) simulations of reaching movements that confirm those criteria, (iii) simulations of reaching movements with inaccurately modeled plants, subject to delays and time-varying feedback and observer gains, and (iv) overshooting and undershooting analysis of those simulations. Time-varying feedback and observer gains are optimal when the cost function involves time-dependent cost matrices, and in particular, when the cost matrices change at the desired reaching time.

A single-joint movement, such as flexing the elbow that was investigated in the dysmetria study in [23], is considered. For simplicity, the rotational movement is approximated by a translational movement, as in [3]. The forearm and hand are modeled as a damped point-mass (with mass *m*_*PL*_ and damping ratio *γ*_*PL*_) to account for viscous damping at the elbow and the damping effect of any external device operated by the hand (e.g., exoskeleton [23], or joystick [24]). Stiffness is not included following [23], which concluded that inertia and damping accounted for much of the observed behavior. Actuation is generated by an over-damped second order muscle model suggested in [3, 25]. The internal model is the same as the actual plant model, except for the values of the mass and damping ratio, *m*_*IM*_ and *γ*_*IM*_, respectively, which may differ from the values *m*_*PL*_ and *γ*_*PL*_ of the plant. The matrices defining the cost function, Eq (8), and the co-variances of the measurement and process noise were taken from [3] or motivated by [26]. The model is detailed in Plant model.

### Theoretical stability analysis

Theoretical stability analysis is conducted for LTI systems, i.e., LTI plants with time-invariant observer and feedback gains. Time-invariant feedback gains result from optmizing cost functions with time-invariant cost-matrices that penalize deviations from the target in the same way at all times (Eq (8)).

The standard equations for the resulting observer-predictor-controller (OPC) system [27] are briefly described below and detailed in Materials and Methods. Novel stability criteria are derived for periodic IC of inaccurately modeled plants. Lemma 1 focuses on the delay-free case, while Lemma 2 considers the effects of delays. The stability criteria are evaluated for translational reaching movements with the simple hand model mentioned above. The results are compared with corresponding stability criteria for CC, which are provided for completion in Stability of continuous LTI systems.

The dynamics of the LTI plant is described by the system matrix $\bar{A}$ and control matrix $\bar{B}$ (Eq (1)), which may differ from the system matrix *A* and control matrix *B* of the internal model (Eq (2)):

$$\begin{array}{c} \dot{x}\left(t\right)=\bar{A}x\left(t\right)+\bar{B}u\left(t\right)+w\left(t\right) \end{array}$$ (1)

$$\begin{array}{c} {\dot{x}}_{IM}\left(t\right)=Ax_{IM}\left(t\right)+Bu\left(t\right) \end{array}$$ (2)

where *x* ∈ *R*^*n*^ is the state of the plant, *u*(*t*) ∈ *R*^*m*^ is the control signal, *w*(*t*) ∈ *R*^*n*^ is the process noise, and *x*_*IM*_ ∈ *R*^*n*^ is the state of the internal model.

The compound effect of process and measurement delay is accounted for by introducing measurement delay *τ*:

$$\begin{array}{c} y(t)=Cx(t-\tau )+v(t-\tau ) \end{array}$$ (3)

where *y*(*t*) ∈ *R*^*q*^ is the measurement and *v*(*t*) ∈ *R*^*q*^ is the measurement noise. We also analyzed the effect of process delay and verified that the stability analysis is the same. Growing evidence suggests that HMC is subject to signal-dependent process and measurement noise [3, 4]. However, for simplicity, both are assumed to be white Gaussian noise with time-independent covariance matrix *W* and *V*, respectively (as in the examples in [27]).

As detailed in Continuous control, the observer generates the delayed estimated state, $\hat{x}\left(t-\tau \right)$, by combining the internal model and the delayed measurement *y*(*t*) according to the observer gain *L*(*t*) (Eq (9)). When *τ* > 0, a predictor is required to predict the current state *x*_*p*_(*t*) (Eq (10), left panel of Fig 1). The controller generates the control signal from either the estimated state (in delay-free systems, when *τ* = 0) or the predicted state (in time-delayed systems, when *τ* > 0), given the feedback gain *K*(*t*) (Eq (11)).

IC performs predictions only at discrete times *t*_*m*_ (right panel of Fig 1). The predictor generates *x*_*p*_(*t*_*m*_), which provides the initial condition for the hold state, *x*_*h*_(*t*). Between samples, the hold state evolves continuously according to the SMH, Eq (13), captured by the feedback matrix:

$$\begin{array}{c} A_{F}\left(t\right)=A-BK\left(t\right) \end{array}$$ (4)

Finally, the controller determines the control signal from the hold state (Eq 12). The evolution and reset of the hold state during IC of reaching movements with accurately and inaccurately modeled plants are illustrated in S1 File. The resulting control signal is also depicted and compared to the control signal generated by CC, to clarify the differences between the two control methods.

#### Periodic IC: Delay-free systems

The control of delay-free systems does not require a predictor, so the dynamics of the system depends only on the state of the plant and the state of the observer, captured by $x_{ov}\left(t\right)\equiv {\left[x{\left(t\right)}^{\prime }\;\hat{x}{\left(t\right)}^{\prime }\right]}^{\prime }$. In periodic IC, the state of the observer is sampled at *t*_*m*_ = *mh*, where *h* is the sampling period. Thus, stability depends on the discrete dynamics of *x*_*ov*_[*m*]≡*x*_*ov*_(*t*_*m*_), specified in Lemma 1.

**Lemma 1**. The discrete dynamics of delay-free LTI systems with periodic IC and sampling period *h* is given by *x*_*ov*_[*m* + 1] = *A*_*p*_*x*_*ov*_[*m*] + *w*_*ov*_[*m*] where *w*_*ov*_[*m*] is the discrete noise term,

$$\begin{array}{c} \begin{array}{cc} A_{p} & =e^{A_{o}h}\left(I_{2n}-S[\begin{array}{cc} 0 & I_{n} \end{array}]\right), \end{array} \end{array}$$ (5)

$$\begin{array}{c} A_{o}=(\begin{array}{cc} \bar{A} & 0\\ LC & A-LC \end{array}),B_{o}=(\begin{array}{c} \bar{B}\\ B \end{array}), \end{array}$$ (6)

and *S* is the solution of the Sylvester equation *γS* + *Sβ* + *α* = 0 with

$$\alpha =(e^{-A_{o}h}B_{o}Ke^{A_{F}h}-B_{o}K),\;\beta =-A_{F},\;\gamma =A_{o}.$$

The proof, detailed in S2 File, is based on solving the dynamics of *x*_*ov*_(*t*), specified by Eq (14)), in continuous time, discretizing the solution at period *h*, and using the SMH (Eq (13)) to determine the hold state and resulting control signal between samples (Eq (12)). A similar approach was used in the networked system design [12], though here we rely on Sylvester equation to derive the discrete system dynamics.

From Lemma 1, the stability of delay-free IC systems with sampling period *h* can be determined from the eigenvalues of *A*_*p*_ specified in Eq (5). Since this is a discrete system, asymptotic stability is guaranteed when all the eigenvalues are inside the unit circle and is lost otherwise.

Lemma 1 was used to evaluate the stability of translational reaching movements with the muscle actuated hand model detailed in Plant model. This model includes 4 state variables (position, velocity, the force generated by the muscle and an internal state of the muscle), so *A*_*p*_ (Eq (5)) has 8 eigenvalues. The observer and feedback gain matrices *L* and *K* were optimized as if the plant is modeled accurately (see Materials and methods for more details). The effect of measurement noise was evaluated by considering both the nominal measurement noise (with the nominal covariance matrix *V* = *V*_0_ detailed in Plant model) and less noisy measurements with *V* = *V*_0_/10.

**Fig 2:**
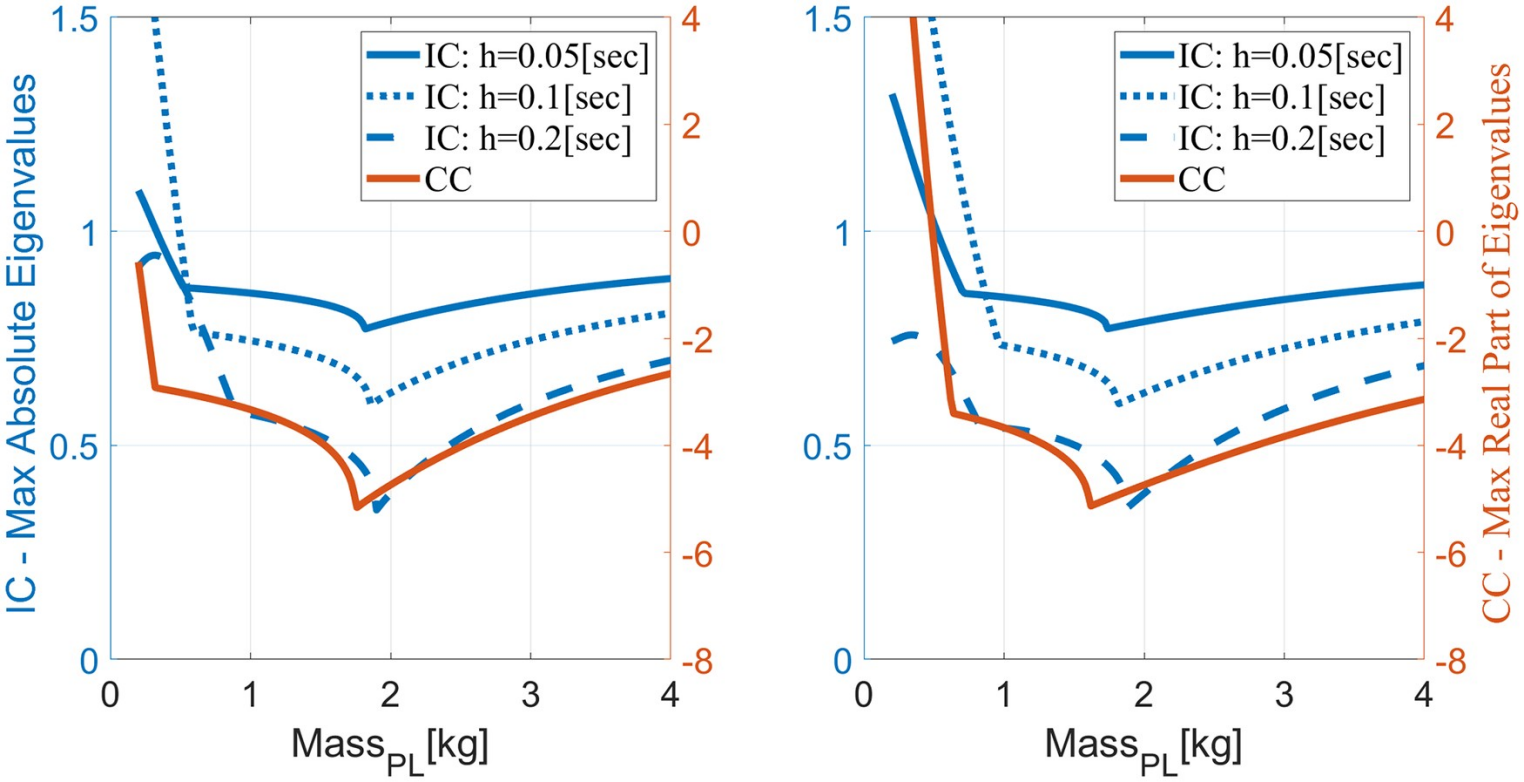
Eigenvalue analysis for reaching movements with LTI plant, observer and controller under IC with different *h* (blue lines) or CC (red line) when *m*_*IM*_ = 2[Kg] as a function of *m*_*PL*_ ∈ [0.2, 4][Kg]. Left y-axis: intermittent control (IC). Right y-axis: continuous control (CC). Left panel: nominal measurement noise with covariance matrix *V* = *V*_0_. Right panel: less noisy measurements with *V* = *V*_0_/10. IC results in a discrete system, so stability is lost when the maximal absolute eigenvalue is larger than one, while CC results in a continuous system so stability is lost when the maximal real part of the eigenvalues is positive. Those two thresholds are aligned to facilitate comparison.

Considering movements of a forearm and hand of mass *m* = 1.5[Kg], we evaluated the eigenvalues of *A*_*ov*_ for *m*_*IM*_ = 2[*Kg*], to account also for the load. For completion, eigenvalues were evaluated for *m*_*PL*_ ∈ (0.2, 4)[Kg], but for normal HMC only the range *m*_*PL*_ ≥ 1.5[Kg] is relevant. Fig 2 depicts the maximum absolute eigenvalue as a function of *m*_*PL*_, for different sampling periods *h* (different blue lines, plotted with respect to the left y-axis), and two covariance matrices for the measurement noise (left panel: *V* = *V*_0_, right panel: *V* = *V*_0_/10). Non-smooth changes in the maximum absolute eigenvalue occur at the transition between different dominant eigenvalues.

**Fig 3:**
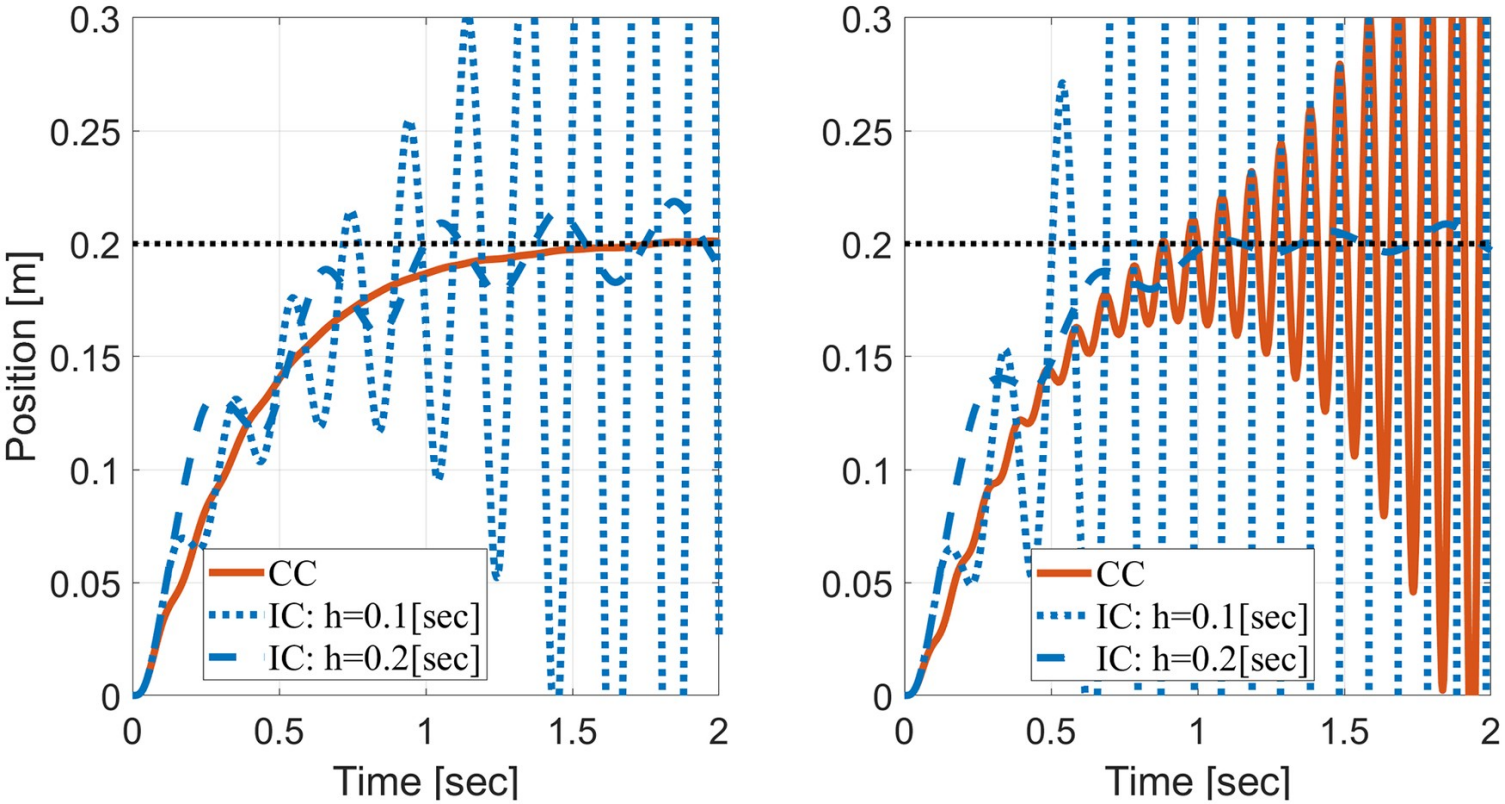
Simulations of reaching movements to demonstrate the stability analysis of Fig 2 for *m*_*IM*_ = 2[Kg] and *m*_*PL*_ = 0.4[Kg]. Left panel: nominal measurement noise with covariance matrix *V* = *V*_0_. Right panel: less noisy measurements with *V* = *V*_0_/10.

For heavier than expected mass (*m*_*PL*_ > *m*_*IM*_) the system remains stable independent of the sampling period *h* and measurement noise. For lighter than expected mass with *V* = *V*_0_ (left panel), stability is lost for *m*_*PL*_ < 0.34[Kg] or *m*_*PL*_ < 0.5[Kg] when *h* = 0.05[sec] or *h* = 0.1[sec], respectively, but is maintained throughout the investigated range when *h* = 0.2[sec]. With less noisy measurements (*V* = *V*_0_/10, right panel), stability is lost for *m*_*PL*_ < 0.54[Kg] or *m*_*PL*_ < 0.78[Kg] when *h* = 0.05[sec] or *h* = 0.1[sec], respectively, but is again maintained when *h* = 0.2[sec]. These results were verified in simulations of translational reaching movements to a target 0.2[m] away. Fig 3 depicts simulated trajectories with *m*_*IM*_ = 2[Kg] and *m*_*PL*_ = 0.4[Kg], when *h* = 0.1[sec] or *h* = 0.2[sec]. In agreement with the stability analysis in Fig 2, IC is stable when *h* = 0.2[sec] but is unstable for the shorter sampling interval *h* = 0.1[sec]. This holds with either the nominal measurement noise (*V* = *V*_0_, left panel) or less noisy measurements (*V* = *V*_0_/10, right panel).

Stability analysis of CC was performed for comparison based on the eigenvalues of *A*_*ov*_, Eq (15), which specifies the dynamics of *x*_*ov*_(*t*) in continuous time, as detailed in Stability of continuous LTI systems. The stability of continuous systems is lost when the real part of any of the eigenvalues become positive. The maximum real part of the 8 eigenvalues of *A*_*ov*_ is plotted on the corresponding panel of Fig 2 with respect to the right y-axis. Note that the two y-axes are aligned so the same y-level line marks the stability thresholds for both systems (unity on the left y-axis, and zero for the right y-axis). CC remains stable throughout the evaluated range when *V* = *V*_0_, but become unstable when *V* = *V*_0_/10 and *m*_*PL*_ < 0.5[Kg], as verified by simulations in Fig 3.

We note that the range of stability becomes narrower when the measurements are less noisy, even though in that case the observer relies more on the measurements and less on the internal models, which may be inaccurate (i.e., the observe gains *L* are larger). The narrower stability range may be attributed to the over-reaction to the unexpected measurements.

#### Periodic IC: Time-delayed systems

The control of time-delayed systems involves a predictor that predicts the current state based on the estimation of the delayed state provided by the observer [27, 28]. In periodic IC of time-delayed systems, the predictor samples the observer at *t*_*m*_ ≡ *mh*, receives $\hat{x}\left[m\right]\equiv \hat{x}\left(t_{m}-\tau \right)$, and generates *x*_*p*_[*m*]≡*x*_*p*_(*t*_*m*_) (right panel of Fig 1). Considering the case where *τ* < *h*, the overall dynamics of the resulting OPC system depends on *x*_*tot*_[*m*] = [*x*_*ov*_[*m*]′ *x*_*p*_[*m* − 1]′]′, as specified in Lemma 2.

**Lemma 2**. The discrete dynamics of LTI systems with delay *τ* under periodic IC with sampling period *h* > *τ* is given by *x*_*tot*_[*m* + 1] = *A*_*tot*_ *x*_*tot*_[*m*] + *w*_*tot*_[*m*], where

$$\begin{array}{c} A_{tot}=(\begin{array}{cc} e^{A_{o}h}\left(I_{2n}-S_{3}e^{-A_{F}\tau }e^{A\tau }\left[0_{n}\;I_{n}\right]\right) & e^{A_{o}h}\left(-S_{2}e^{-A_{F}\tau }+S_{3}e^{-A_{F}\tau }S_{1}\right)e^{A_{F}h})\\ e^{A\tau }\left[0_{n}\;I_{n}\right] & -S_{1}e^{A_{F}h} \end{array}),\; \end{array}$$ (7)

*A*_*o*_ is defined by Eq (6), *A*_*F*_ is defined by Eq (4), and *S*_*i*_, *i* = 1, 2, 3 are solutions to three Sylvester equations *γ*_*i*_ *S*_*i*_ + *S*_*i*_ *β*_*i*_ + *α*_*i*_ = 0 with

$$\begin{array}{c} \alpha_{1}=BK-e^{A\tau }BKe^{-A_{F}\tau },\;\beta_{1}=-A_{F},\;\gamma_{1}=A,\; \end{array}$$

$$\begin{array}{c} \alpha_{2}=e^{-A_{o}\tau }B_{c}Ke^{A_{F}\tau }-B_{o}K,\;\beta_{2}=-A_{F},\;\gamma_{2}=A_{o},\; \end{array}$$

$$\begin{array}{c} \alpha_{3}=e^{-A_{o}h}B_{c}Ke^{A_{F}h}-e^{-A_{o}\tau }B_{c}Ke^{A_{F}\tau },\;\beta_{3}=-A_{F},\;\gamma_{3}=A_{o}.\; \end{array}$$

The proof, detailed in S2 File, is based on developing a difference equation for *x*_*p*_[*m*] that depends on $\hat{x}\left[m\right]$ and a difference equation for *x*_*ov*_[*m*] that depends on *x*_*p*_[*m*] and *x*_*p*_[*m* − 1]. The dynamics of *x*_*tot*_[*m*] is derived by combining those two difference equations.

From Lemma 2, the stability of time-delayed systems with periodic IC can be determined from the eigenvalues of *A*_*tot*_ specified in Eq (7). Since this is a discrete system, asymptotic stability is guaranteed when all the eigenvalues are inside the unit circle, and is lost otherwise.

**Fig 4:**
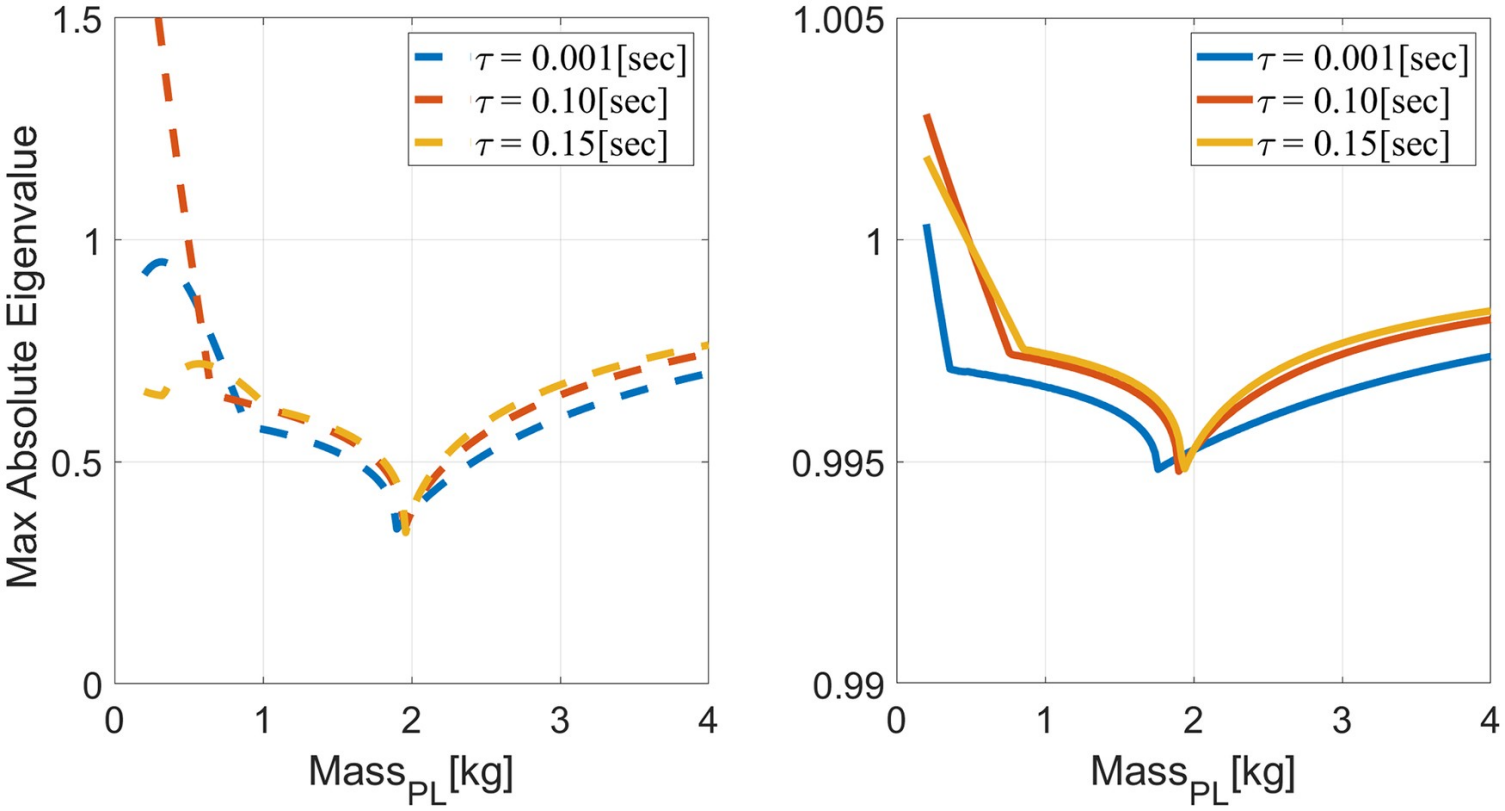
Eigenvalue analysis of reaching movements with LTI plant, observer and controller, for *m*_*IM*_ = 2[Kg] as a function of *m*_*PL*_ ∈ [0.2, 4][Kg]. Left panel: periodic IC with *h* = 0.2[sec]. Right panel: discrete equivalent system of CC with Δ = 10^−3^. In both cases stability is lost when the maximal absolute eigenvalue is larger than one.

**Fig 5:**
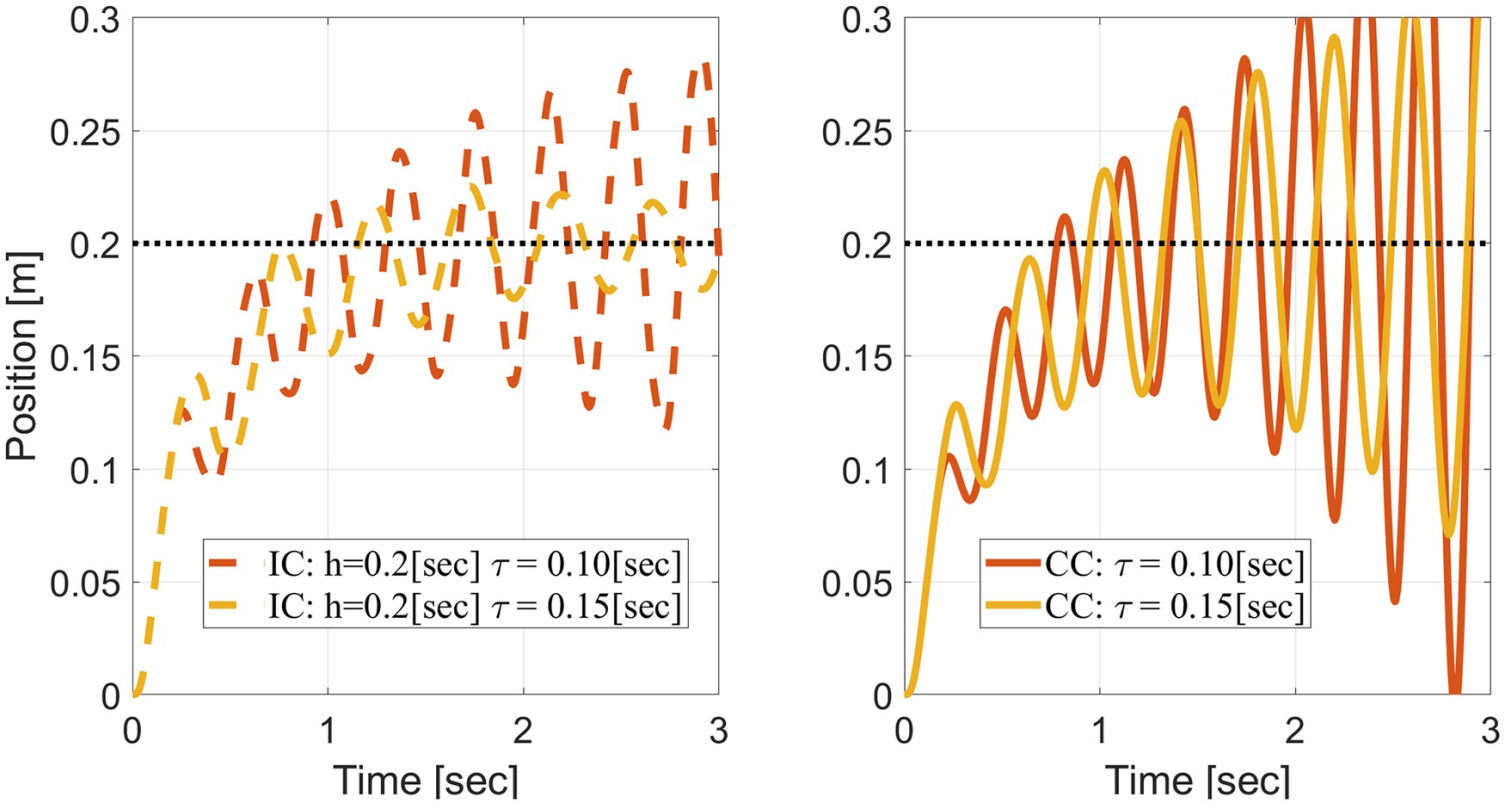
Simulations of reaching movements to demonstrate the stability analysis of Fig 4 for *m*_*IM*_ = 2[Kg] and *m*_*PL*_ = 0.4[Kg]. Left panel: periodic IC with *h* = 0.2[sec]. Right panel: discrete equivalent system of CC with Δ = 10^−3^.

The left panel of Fig 4 depicts the maximum absolute eigenvalue of *A*_*tot*_ when *V* = *V*_0_, *h* = 0.2[sec], and *m*_*IM*_ = 2[*Kg*], as a function of *m*_*PL*_ ∈ [0.2, 4][Kg], for different delays *τ* < *h*. A delay of *τ* = 0.1[sec] causes the system to become unstable when *m*_*PL*_ < 0.5[Kg]. Interestingly, the system remains stable over all the investigated range with a longer delay of *τ* = 0.15[sec] (see also first row of Table 1). These results were verified in simulations, as depicted in the left panel of Fig 5 for reaching movements with *m*_*IM*_ = 2[Kg] and *m*_*PL*_ = 0.4[Kg]. In agreement with the stability analysis, IC is stable when *τ* = 0.15[sec] and unstable for shorter delay of *τ* = 0.1[sec].

**Table 1:** Stability threshold: Minimum *m*_*PL*_[Kg] for which reaching movements with LTI plant, observer and controller are stable under CC or IC with *h* = 0.2[sec].

*m*_*IM*_	*γ*_*IM*_	IC	IC	CC	CC
[Kg]	[Nsec/m]	*τ* = 0.1[sec]	*τ* = 0.15[sec]	*τ* = 0.1[sec]	*τ* = 0.15[sec]
2	8	0.5	< 0.2	0.5	0.48
4	8	< 0.4	1.24	0.88	1.04
6	8	1.5	1.98	1.2	1.5
2	6	0.56	< 0.2	0.62	0.66
4	6	1.0	1.56	1.08	1.24
6	6	1.86	2.28	1.44	1.8

Stability of time-delayed systems under CC was analyzed for comparison. The analysis was facilitated by converting the time-delayed continuous system to an equivalent time-delayed discrete-time system using the standard zero-order hold (ZOH), as detailed in Stability of continuous LTI systems. The equivalent discrete-time system is stable as long as all the eigenvalues of *A*_*ex*_, specified in Eq (16), are inside the unit circle. Note that an eigenvalue λ_*d*_ of a discrete-time system depends on the discretization time Δ, and evolves as $\lambda_{d}^{k}$ at *t* = *k*Δ. For comparison with the eigenvalues of IC, consider the evolution of λ_*d*_ after *t* = *h* = 0.2[sec]. Here we used Δ = 10^−3^, so λ_*d*_ = 1.001, for example, would evolve to 1.001^200^ = 1.22.

The right panel of Fig 4 depicts the maximum absolute eigenvalue of *A*_*ex*_, indicating that time-delayed systems with *τ* = 0.001/0.1/0.15[sec] become unstable when *m*_*PL*_ < 0.22/0.5/0.48[Kg], respectively (first row in Table 1). These results were verified in simulations, as depicted in the right panel of Fig 5 for reaching movements with *m*_*IM*_ = 2[Kg] and *m*_*PL*_ = 0.4[Kg]. In agreement with the stability analysis, CC is unstable with both *τ* = 0.1[sec] and *τ* = 0.15[sec].

The stability analysis in Fig 4 was repeated for different *m*_*IM*_ and *γ*_*IM*_, and the resulting stability thresholds are listed in Table 1. Neither CC nor IC is consistently superior to the other in terms of the range of loads over which the system remains stable. CC is superior when *m*_*IM*_ = 6[Kg], while IC is superior when *m*_*IM*_ = 4[Kg] and *τ* = 0.1[sec] or *m*_*IM*_ = 2[Kg] and *τ* = 0.15[sec]. The effect of the delay is also inconsistent. For both *m*_*IM*_ = 4[Kg] and *m*_*IM*_ = 6[Kg], a system that is stable with *τ* = 0.15[sec] remains stable when the delay is shortened to *τ* = 0.1[sec], but this is not the case when *m*_*IM*_ = 2[Kg], as indicated by Figs 4 and 5.

### Reaching movements with time-varying gains

Human reaching is performed under time constraints that can be optimally satisfied with time-dependent feedback and observer gains [6, 29]. This hinders theoretical analysis, so we investigate the effects of plant inaccuracies using numerical simulations. The simulated task was to reach a target located 0.2[m] away from the initial position at *T* = 0.6[sec], with the hand model detailed in Plant model and measurement delay of *τ* = 0.15[sec]. Qualitative aspects of the responses are compared with previously published experimental results, both with ataxia patients and with healthy participants subjected to perturbations in the inertia and damping of the manipulated exoskeleton [23].

#### Reaching movements with inaccurately modeled plants

**Fig 6:**
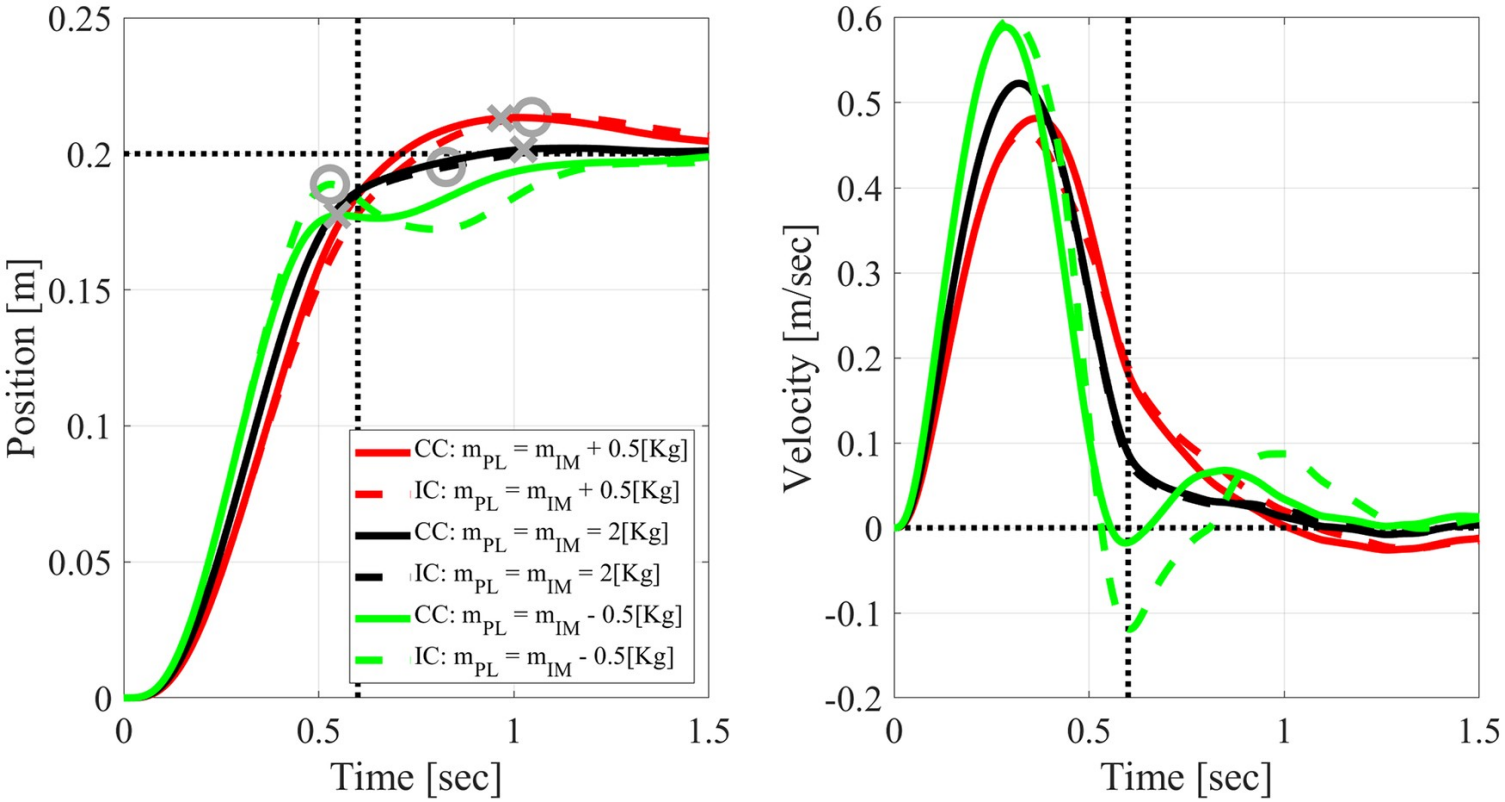
Effect of inaccurately modeled mass on simulated reaching movements with measurement delay *τ* = 0.15[sec]. Solid: CC. Dashed: periodic IC with *h* = 0.2[*sec*]. The mass of the plant, *m*_*PL*_, is either accurate (black), lighter (green), or heavier (red) than the mass of the internal model, *m*_*IM*_ = 2[*Kg*]. Left: position. Right: velocity. Vertical dashed lines mark the desired duration of movement, *T* = 0.6[sec]. Horizontal dashed line marks the target position. Crosses and circles mark the positions from which dysmetria was evaluated for CC and IC, respectively (see text).

**Fig 7:**
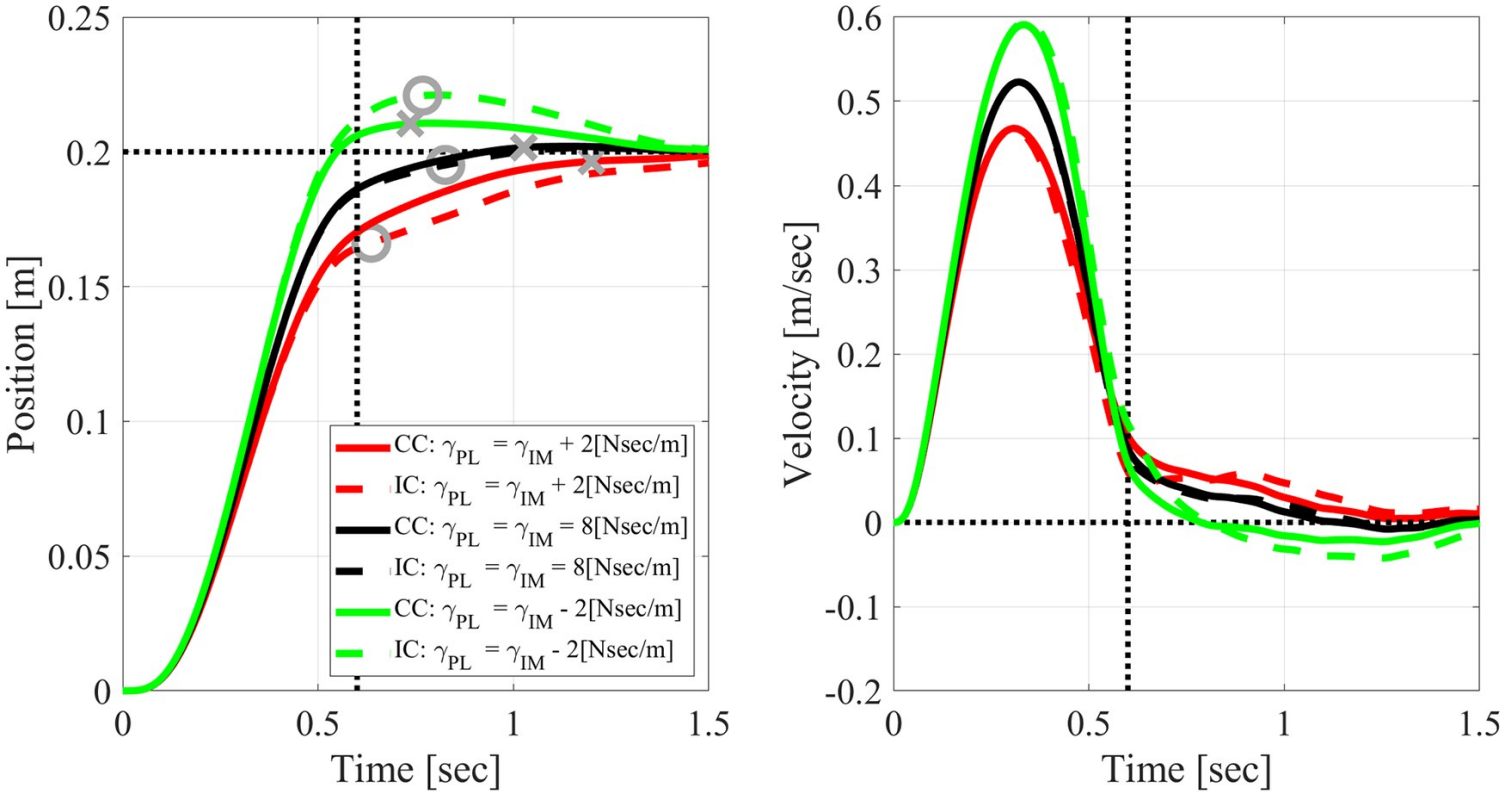
Effect of inaccurately modeled damping ratio on simulated reaching movements with measurement delay of *τ* = 0.15[sec]. Solid: CC. Dashed: periodic IC with *h* = 0.2[*sec*]. The damping ratio of the plant, *γ*_*PL*_, is either smaller (green), equal (black) or larger (red) than the damping ratio of the internal model, *γ*_*IM*_ = 8[*Nsec*/*m*]. Left: position. Right: velocity. Dashed lines, circles and crosses as in Fig 6.

Figs 6 and 7 compare the trajectories generated by CC (solid lines) versus periodic IC (with *h* = 0.2[sec], dashed lines) when reaching with inaccurately modeled mass or damping, respectively. The parameters of the internal model were kept constant at the nominal values *m*_*IM*_ = 2[Kg] and *γ*_*IM*_ = 8[Nsec/m]), while the parameters of the actual plant, *m*_*PL*_, and *γ*_*PL*_, were smaller, the same, or larger than expected.

Both figures demonstrate that when the internal model is accurate, the responses generated by CC and by periodic IC are very similar, even though the sampling period, *h* = 0.2[sec], is third of the desired reaching time *T* = 0.6[sec]. This can be attributed to the accuracy of the SMH (Eq (13)) that IC uses to approximate the predicted state between samples. When the plant differs from the internal model, the responses generated by CC and IC deviate from each other, though the main characteristics remain the same. In particular, both controllers overshoot the target when the plant is heavier or the damping is smaller than expected. The effect of the mass may seem counter-intuitive, but agrees well with experimental results and other models [23]. When the plant is lighter than expected, i.e., *m*_*PL*_ = *m*_*IM*_ − 0.5[Kg] (Fig 6), both CC and IC result in oscillatory trajectory, which are more pronounced with IC.

#### Overshooting and undershooting analysis

Overshooting or undershooting the target, known also as hypermetria and hypometria, respectively, are typical aspects of dysmetria. Following a study of motor dysmetria in ataxia patients during elbow movements, we quantified dysmetria by the difference between the target position and the position at the time of first correction [23]. The latter was defined as the first time the velocity decreased below a threshold of 0.01[m/sec], or reached a local minimum after the peak velocity. The positions from which dysmetria was computed are marked by crosses (CC) or circles (IC) in Figs 6 and 7. It is evident that inaccurately modeled mass and damping have opposite effects on dysmetria according to both CC and periodic IC. In particular, while a lighter/heavier than expected plant results in undershoot/overshoot (Fig 6), a smaller/larger than expected damping ratio results in overshoot/undershoot (Fig 7).

**Fig 8:**
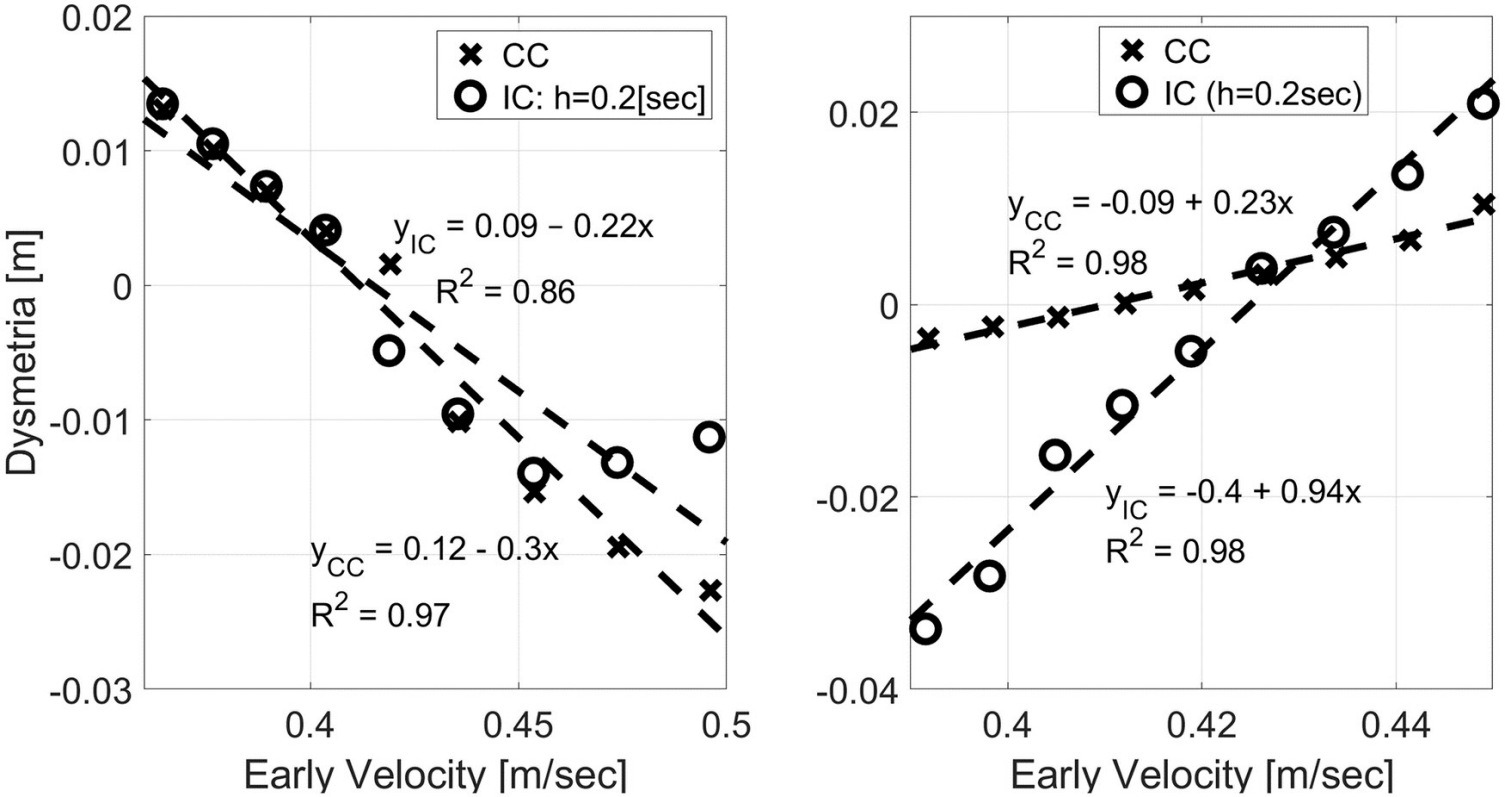
Dysmetria versus early velocity from simulations with inaccurate mass (left) or inaccurate damping ratio (right) for *m*_*IM*_ = 2[Kg] and *γ*_*IM*_ = 8[Nsec/m]. All simulations were conducted with *τ* = 0.15[sec]. IC was conducted with *h* = 0.2[sec]. Mass (left): *m*_*PL*_ = (1.5, 2.5)[Kg] evenly spaced around *m*_*IM*_. Damping ratio (right): *γ*_*PL*_ ∈ (6, 10)[Nsec/m] evenly spaced around *γ*_*IM*_].

Following [23], we evaluated the dependence of dysmetria on early velocity, which reflects the preplanned control before feedback driven corrections. Early velocity was defined as the velocity 0.15[sec] after movement onset, i.e., after the velocity exceeded 0.05[m/sec]. The analysis in Figs 6 and 7 was repeated for *N* = 9 different values of *m*_*PL*_ ∈ (1.5, 2.5)[Kg] evenly spaced around *M*_*IM*_ = 2[Kg] and separately for *N* = 9 different values of *γ*_*PL*_ ∈ (6, 10)[Nsec/m] evenly spaced around *γ*_*IM*_ = 8[Nsec/m]. Dysmetria and early velocity were extracted from each simulation and plotted against each other in Fig 8. Under both CC and periodic IC, dysmetria and early velocity are negatively correlated when reaching with inaccurately modeled mass (left panel, Fig 8), and positively correlated when reaching with inaccurately modeled damping ratio (right panel, Fig 8). Those correlations are in agreement with experimental results with healthy subjects [23], and the feedforward / feedback computational model suggested there.

Discrepancies between CC and IC in the accurate case (*m*_*PL*_ = *m*_*IM*_ and *γ*_*PL*_ = *γ*_*IM*_) are due to the local velocity minima that emerges in the trajectories of IC at this case. Thus, the position of first correction, from which dysmetria was calculated, had been determined by different criteria: velocity decreasing below 0.01[m/sec] in CC and local velocity minima in IC. The relationship between dysmetria and early velocity is highly linear, as evident in the linear regression results listed on the graphs, except when *m*_*PL*_ is much lower than *m*_*IM*_. Deviations from linearity in this range can be attributed to the early peak in the position that occurs when *m*_*PL*_ is much lower than *m*_*IM*_, as is evident in Fig 6 for *m*_*PL*_ = 1.5[Kg].

**Fig 9:**
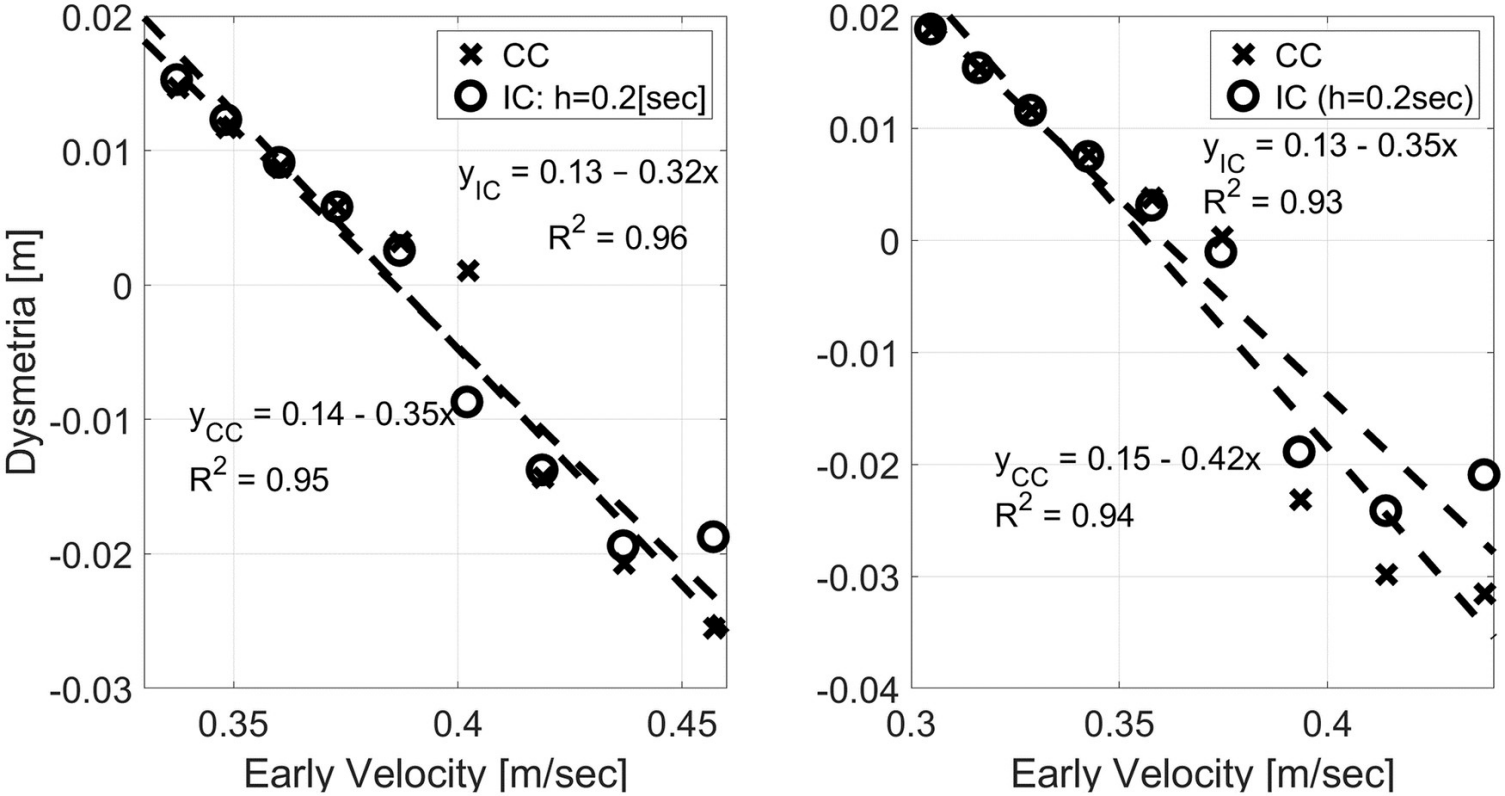
Dysmetria versus early velocity from simulations with inaccurate mass and modified cost function when *m*_*IM*_ = 2[Kg] (left) and *m*_*IM*_ = 4[Kg] (right). All simulations were conducted with *τ* = 0.15[sec] and *Q*_∞_ = *diag*(1, 0.1^2^, 0.02^2^, 0) instead of the nominal *Q*_∞_ = *diag*(1, 0.2^2^, 0.02^2^, 0). IC was conducted with *h* = 0.2[sec]. Left: *m*_*PL*_ = (1.5, 2.5)[Kg] evenly spaced around *m*_*IM*_ = 2[Kg]. Right: *m*_*PL*_ = (3, 5)[Kg] evenly spaced around *m*_*IM*_ = 4[Kg].

The negative correlation between dysmetria and early velocity is of particular interest since it was observed across a group of ataxia patients [23]. Fig 9 demonstrates this negative correlation for two other sets of parameters. In both cases, the cost function was modified to reduce the penalty on deviations from the desired null velocity for *t* > *T*, with *Q*_∞_ = *diag*(1, 0.1^2^, 0.02^2^, 0) instead of the nominal *Q*_∞_ = *diag*(1, 0.2^2^, 0.02^2^, 0). The left and right panels depict the dysmetria analysis for *m*_*IM*_ = 2[Kg] and *m*_*IM*_ = 4[Kg], respectively. With the modified penalty, the correlation between dysmetria and early velocity under IC is more linear (left panel of Fig 8 versus the left panel of Fig 9). This can be attributed to the smaller initial peak in the position when *m*_*PL*_ is much lower than *m*_*IM*_.

## Materials and methods

### Cost function

Optimal control minimizes a cost function *J* that usually penalizes for deviations from the desired state and for the control effort. Stable linear systems converge to the origin, which is redefined to be the target state by change of variables (e.g., position is measured from the target position). Thus deviations from the origin are just the norm of the state vector, which can be weighted and computed in different ways. A standard cost function, which facilitates analysis, is the quadratic cost function, based on the weighted quadratic norm [6]:

$$\begin{array}{c} J=E[x^{\prime }\left(T\right)S\left(T\right)x\left(T\right)+\int_{0}^{T}\left(x^{\prime }\left(t\right)Q\left(t\right)x\left(t\right)+u^{\prime }\left(t\right)R\left(t\right)u\left(t\right)dt\right)] \end{array}$$ (8)

where *T* is the desired time to reach the target, also known as terminal time or horizon. When deviations from the target state are important at all times, the cost function in Eq (8) is extended to the infinite horizon case by taking the limit *T* → ∞.

The first term in the integral penalizes for deviations of the state from the origin weighted by a positive semidefinite matrix *Q*(*t*). The diagonal terms in this matrix are the weights of the square magnitude of the corresponding state variables, while other terms are usually set to zero. The second term in the integral penalizes for the square magnitude of the effort weighted by a positive definite matrix *R*(*t*). When the control signal is scalar, as in our case, *R*(*t*) is a positive scalar function of time. The term outside the integral penalizes for deviations from the origin at the (finite) terminal time, weighted by a positive semidefinite matrix *S*(*T*).

When *T* is finite, the cost function does not constrain the feedback gains for *t* > *T*. Nevertheless, those gains are important when the target is not reached at *t* = *T*, as is usually the case when controlling inaccurately modeled plants. Here we extend the gains at *t* = *T* to *t* > *T*, and assure that they do not vanish by proper selection of *S*(*T*). As detailed below, *S*(*T*) is selected so this is equivalent to optimizing the infinite horizon cost function, with *Q*(*t* > *T*) = *Q*_∞_ and *Q*(*t* < *T*) = *Q*(*t*) (see Plant model).

### Continuous control

Optimizing Eq (8) involves two sub-processes: observer that estimates the state and controller that generates the control signal from the estimated state [6]. As mentioned above, we simplify the analysis by assuming that the noise is signal independent white Gaussian noise, despite physiological evidence that the noise might be signal-dependent [3, 4]. Under this simplifying assumption, the two sub-processes can be optimized independent of each other. When there are delays in the system, as in HMC, a third intermediate sub-process, known as the predictor, is required to predict the current state from the delayed estimated state. The control signal in that case is derived from the predicted, rather than the estimated, state. The resulting three sub-processes are also known as the Observer-Predictor-Feedback (OPF) structure [5].

*Observer* combines the internal model (Eq (2)) and delayed measurement (Eq (3)) to generate the estimated state $\hat{x}$ according to [6, 27, 29]:

$$\begin{array}{c} \dot{\hat{x}}\left(t-\tau \right)=A\hat{x}\left(t-\tau \right)+Bu\left(t-\tau \right)+L\left(t\right)\left(y\left(t\right)-C\hat{x}\left(t-\tau \right)\right) \end{array}$$ (9)

where *L*(*t*) is the observer gain matrix. Under the assumption that the internal model is accurate, the optimal observer gain is the Kalman gain *L*(*t*) = *P*(*t*)*C*′ *V*^−1^(*t*) where *P*(*t*) is the solution to the Riccati differential equation $\dot{P}\left(t\right)=AP\left(t\right)+P\left(t\right)A^{\prime }-P\left(t\right)C^{\prime }V^{-1}CP\left(t\right)+W$ [6, 29]. For infinite horizon, the Kalman gain is time-invariant *L*(*V*, *W*) = *P*_∞_ *C*′ *V*^−1^ where *P*_∞_ is the solution of the algebraic Riccati equation 0 = *AP*_∞_ + *P*_∞_ *A*′ − *P*_∞_ *C*′ *V*^−1^ *CP*_∞_ + *W* [6, 29].

*Predictor* predicts the current state, *x*_*p*_(*t*), given the estimated state, $\hat{x}\left(t-\tau \right)$, and the control signal *u*(*σ*) for *σ* ∈ [*t* − *τ*, *t*), based on the internal model (Eq (2)):

$$\begin{array}{c} x_{p}\left(t\right)=e^{A\tau }\hat{x}\left(t-\tau \right)+\int_{t-\tau }^{t}e^{A(t-\sigma )}Bu\left(\sigma \right)d\sigma \end{array}$$ (10)

*Controller* generates the control signal *u*(*t*) given the predicted state:

$$\begin{array}{c} u\left(t\right)=-K\left(t\right)x_{p}\left(t\right) \end{array}$$ (11)

where *K*(*t*) is the feedback gain matrix. For infinite horizon with time-invariant cost matrices (*Q*(*t*) = *Q*_∞_ and *R*(*t*) = *R*_∞_), the optimal feedback gain is time-invariant $K=K_{\infty }\left(Q_{\infty },R_{\infty }\right)\equiv R_{\infty }^{-1}B^{\prime }S_{\infty }$ where *S*_∞_ is the solution of the algebraic Riccati equation $0=A^{\prime }S_{\infty }+S_{\infty }A-S_{\infty }BR_{\infty }^{-1}B^{\prime }S_{\infty }+Q_{\infty }$ [6, 29]. For finite terminal time, the optimal feedback gain matrix is *K*(*t*) = *R*^−1^(*t*)*B*′ *S*(*t*) where *S*(*t*) is the solution to the Riccati differential equation: $-\dot{S}\left(t\right)=A^{\prime }S\left(t\right)+S\left(t\right)A-S\left(t\right)BR{\left(t\right)}^{-1}B^{\prime }S\left(t\right)+Q\left(t\right)$ [6, 29] that is solved backward, starting with *S*(*T*). Due to plant inaccuracies, the plant may not reach the desired state at *t* = *T*, so feedback gains are relevant past the finite terminal time. For continuity, *K*(*t* > *T*) = *K*(*T*), and by setting *S*(*T*) = *S*_∞_(*Q*_∞_, *R*_∞_), *K*(*T*) = *K*_∞_(*Q*_∞_, *R*_∞_). This is equivalent to optimizing an infinite horizon cost funtion with *Q*(*t* > *T*) = *Q*_∞_ and *Q*(*t* < *T*) = *Q*(*t*).

### Intermittent control

IC performs predictions at discrete times *t*_*m*_, which can be evenly spaced (periodic IC, *t*_*m*_ = *mh*, where *h* is the sampling period) or event driven (not considered in this work). At *t*_*m*_, the predictor receives $\hat{x}\left(t_{m}-\tau \right)$ from the observer and generates *x*_*p*_(*t*_*m*_) according to Eq (10). The latter provides the initial condition for the hold state, *x*_*h*_(*t*) that determines the control signal:

$$\begin{array}{c} u\left(t\right)=-K\left(t\right)x_{h}\left(t\right) \end{array}$$ (12)

Between samples, *x*_*h*_(*t*) evolves continuously according the feedback matrix (Eq (4)), defining the SMH [5]:

$$\begin{array}{c} \left\{ \begin{array}{c} {\dot{x}}_{h}\left(t\right)=A_{F}\left(t\right)x_{h}\left(t\right),\;t\in \left[t_{m-1},t_{m}\right)\\ x_{h}\left(t_{m}^{+}\right)=x_{p}\left(t_{m}\right),\;\forall m\in Z^{+} \end{array}\right. \end{array}$$ (13)

### Stability of continuous LTI systems

Stability is analyzed for LTI systems with time-invariant observer and controller gains, *L* and *K*. In that case, Eqs (1), (3) and (9) can be combined to describe the LTI dynamics of the overall state $x_{ov}\left(t-\tau \right)={\left[x{\left(t-\tau \right)}^{\prime }\;\;\hat{x}{\left(t-\tau \right)}^{\prime }\right]}^{\prime }$:

$$\begin{array}{c} {\dot{x}}_{ov}\left(t-\tau \right)=A_{o}x_{ov}\left(t-\tau \right)+B_{o}u\left(t-\tau \right)+w_{ov}\left(t-\tau \right) \end{array}$$ (14)

where *A*_*o*_ and *B*_*o*_ are defined in Eq (6), and *w*_*ov*_(*t* − *τ*) = [*w*(*t* − *τ*)′ *Lv*(*t* − *τ*)′]′ is the overall process noise.

In the delay-free case, the predicted state is the same as the estimated state. Thus, the control signal is determined from the estimated state given the state feedback gain *K* (Eq (11)), resulting in an autonomous system: ${\dot{x}}_{ov}\left(t\right)=A_{ov}x_{ov}+w_{ov}\left(t\right)$, where

$$\begin{array}{c} A_{ov}=(\begin{array}{cc} \bar{A} & -\bar{B}K\\ LC & A-BK-LC \end{array}). \end{array}$$ (15)

Asymptotic stability is guaranteed as long as the real parts of all the eigenvalues of *A*_*ov*_ are negative, and is lost otherwise.

For systems with delays, stability analysis is facilitated by converting the continuous system to an equivalent discrete-time system, using the standard zero-order hold [6]. The discretization time Δ is selected so *k*_*τ*_ = *τ*/Δ is an integer number. Thus, the overall dynamics of the equivalent discrete-time system depends on *k*_*τ*_ samples of the predicted state. Defining the extended discrete state $x_{ex}\left(k\right)={\left[x{\left(k-k_{\tau }\right)}^{\prime }\;\hat{x}{\left(k-k_{\tau }\right)}^{\prime }\;x_{p}{\left(k-1\right)}^{\prime }\;\ldots \;x_{p}{\left(k-k_{\tau }\right)}^{\prime }\right]}^{\prime }$, S3 File shows that with time-invariant observer and feedback gain matrices, *L*_*d*_ and *K*_*d*_, respectively, *x*_*ex*_(*k* + 1) = *A*_*ex*_ *x*_*ex*_(*k*) + *w*_*ex*_(*k*), where

$$\begin{array}{c} A_{ex}=(\begin{array}{ccccccc} {\bar{A}}_{d} & 0 & 0 & 0 & \cdots & 0 & -{\bar{B}}_{d}K_{d}\\ L_{d}C & \left(A_{d}-L_{d}C\right) & 0 & 0 & \cdots & 0 & -B_{d}K_{d}\\ 0 & A_{d}^{k_{\tau }} & -B_{d}K_{d} & -A_{d}B_{d}K_{d} & \cdots & -A_{d}^{k_{\tau }-2}B_{d}K_{d} & -A_{d}^{k_{\tau }-1}B_{d}K_{d}\\ 0 & 0 & I & 0 & \cdots & 0 & 0\\ 0 & 0 & 0 & I & \cdots & 0 & 0\\ \vdots & \vdots & \vdots & \vdots & \vdots & \vdots & \vdots \\ 0 & 0 & 0 & 0 & \cdots & I & 0 \end{array}) \end{array}$$ (16)

${\bar{A}}_{d}=exp\left(\bar{A}\Delta \right)$, ${\bar{B}}_{d}={\bar{A}}^{-1}\left(exp\left(\bar{A}\Delta \right)-I\right)\bar{B}$, *A*_*d*_ = *exp*(*A*Δ) *B*_*d*_ = *A*^−1^(*exp*(*A*Δ) − *I*)*B*, and *w*_*ex*_(*k*) is the discrete process noise.

Asymptotic stability is guaranteed as long as all the eigenvalues of *A*_*ex*_ are within the unit circle, and is lost otherwise.

### Plant model

As mentioned above, the rotational movement of the hand around the elbow is approximated by a translational movement, as in [3]. The translational position of the hand, *p*_*h*_, in response to the force, *f*, is governed by:

$$\begin{array}{c} m_{PL}\ddot{p_{h}}\left(t\right)+\gamma_{PL}{\dot{p}}_{h}\left(t\right)=f \end{array}$$ (17)

The force is generated by an over-damped second order muscle model with time constants *μ*_1_ = *μ*_2_ = *μ* = 0.04[*sec*]. The force generated by the muscle in response to the control signal *u*(*t*), corrupted by process noise, *w*_*g*_(*t*), is given by:

$$\begin{array}{c} \mu \dot{f}+f=g\left(t\right) \end{array}$$ (18)

$$\begin{array}{c} \mu \dot{g}+g=u\left(t\right)+w_{g}\left(t\right) \end{array}$$ (19)

where *g* is an internal state of the muscle, and the variance of the process noise is $\sigma_{g}^{2}={\left(0.46\left[N\right]\right)}^{2}$ [3].

Defining the hand state $x\left(t\right)={\left[p_{h}\left(t\right)\;{\dot{p}}_{h}\left(t\right)\;f\left(t\right)\;g\left(t\right)\right]}^{\prime }$, Eqs (17)–(19) can be expressed in the state-space representation of Eq (1) with:

$$\begin{array}{c} \bar{A}=(\begin{array}{cccc} 0 & 1 & 0 & 0\\ 0 & -\gamma_{PL}/m_{PL} & 1/m_{PL} & 0\\ 0 & 0 & -1/\mu & 1/\mu \\ 0 & 0 & 0 & -1/\mu \end{array}),\bar{B}=(\begin{array}{c} 0\\ 0\\ 0\\ 1/\mu \end{array}),w\left(t\right)=(\begin{array}{c} 0\\ 0\\ 0\\ w_{g}\left(t\right)/\mu \end{array}) \end{array}$$ (20)

The internal model is the same as the actual plant model, except for the values of the mass and damping ratio, *m*_*IM*_ and *γ*_*IM*_, respectively, which may differ from the values *m*_*PL*_ and *γ*_*PL*_ of the plant.

The average mass of the human forearm and hand is *m* = 1.5[Kg] [30], so we consider *m*_*IM*_ = 2–6[Kg] to account also for the mass of the load. The value of the linear damping ratio *γ*_*hand*_ was motivated by the angular damping ratio *γ*_*θ*_ ∈ (0.5–0.8)[Nmsec/rad] for elbow flexion/extension [31]. Using standard conversion from joint to end-point damping [32], $\gamma_{hand}=\gamma_{\theta }/l_{arm}^{2}$ where *l*_*arm*_, is the length of the forearm. For *l*_*arm*_ ≊ 0.3[m], *γ*_*hand*_ ≊ (5.5 − 9) [Nsec/m]. Thus we use *γ*_*IM*_ = 8[Nsec/m] to account for viscous damping at the elbow and the effect of any external device operated by the hand.

Only the position and velocity of the hand are sensed, so *C* = [*I*_2×2_ 0_2×2_]. The nominal covariance matrix of the measurement noise is $V_{0}=diag\left(\left[\sigma_{p}^{2}\;\sigma_{v}^{2}\right]\right)$, where $\sigma_{p}^{2}={\left(0.005\left[m\right]\right)}^{2}$ and $\sigma_{v}^{2}={\left(0.04\left[m/s\right]\right)}^{2}$ are the variances of the (visual) measurement noise of the position and velocity, respectively. These values were estimated from [26], for mean position error of 0.09[m], and mean velocity of 0.28[m/sec].

The cost function, Eq (8), penalizes for control effort with *R*(*t*) = *R*_∞_ = 0.00001 and for deviations from the target with *Q*(*t*) = *Q*_∞_ = *diag*([1 0.2^2^ 0.02^2^ 0]) [3]. While deviaions in the position are penalized most severely, this cost matrix also penalizes deviations from zero velocity and zero muscle force, which are required for staying at the target position. Finite reaching movements are imposed by setting *Q*(*t* < *T*) = 0_4×4_, to allow for high velocity and force required to reach the target at *T* = 0.6[sec].

## Discussion

Intermittent control (IC) has been advocated as a more viable model for HMC [5, 19, 20] than continuous control (CC). Using system-matched hold (SMH), IC combines short-term open-loop control with intermittent closed-loop feedback to exploit the advantages of both. In particular, IC reduces the communication and computational burden associated with continuous prediction. For these reasons, IC is also gaining interest in the control literature, where it is known as sampled data control (SDC) [11]. Furthermore, IC has been shown to provide better agreement with some HMC experiments as reviewed in the Introduction [5], and to increase robustness to perturbations during quiet and tiptoe standing [33, 34]. However, it is not clear how well IC maintains stability when controlling inaccurately modeled plants.

Our theoretical analysis, supported by numerical simulations, demonstrates that under some conditions, periodic IC may remain stable under a wider range of plant inaccuracies than CC, especially with less noisy measurements (right panels of Figs 2 and 3) or in the presence of delays (Figs 4 and 5). Interestingly, periodic IC with sample period of *h* = 0.2[sec] may remain stable under a wider range of inaccuracies with longer delays (e.g., with a delay of 0.15[sec] compared to a delay of 0.1[sec] or 0.001[sec]). While IC is not consistently superior to CC in terms of the range of loads over which the system remains stable, it is demonstrated to cope comparatively well with plant inaccuracies.

Simulations of reaching movements with time-varying gains, which are more relevant for HMC, demonstrate that periodic IC cope well with mismatches between the internal model and plant dynamics. Compared to CC, IC tends to generate more oscillatory responses and stronger overshoot/ undershoot. Interestingly, human experiments indicate that hypometric patients, whose internal model may not match the actual plant, also generate oscillatory reaching movements. Thus, both theoretical analysis and simulations suggest that IC is indeed a viable candidate for HMC.

Numerical simulations with different mass or damping inaccuracies agree well with previously reported experimental results with healthy participants where inertia and damping perturbations were introduced by an exoskeleton [23]. In particular, both CC and IC result in the observed negative/ positive correlation between dysmetria and early velocity with inaccurately modeled mass/ damping, respectively. Damping inaccuracies result in a stronger effect on periodic IC than on CC, while mass inaccuracies result in a similar effect (left and right panels of Figs 8 and 9, respectively).

Experimental results with patients having cerebellar deficits were shown to be characterized by negatively correlated dysmetria and early velocity [23]. Hence, it was hypothesized that cerebellar dysmetria is related to biased inertia in the internal model [23]. A computational model based on feedforward/ feedback control was suggested to explain the experimental results. The feedforward control signal was derived from the desired trajectory based on the internal model of the plant, and then combined with a feedback control signal (Figure 4 in [23]). The desired trajectory was preplanned to generate a bell-shaped velocity profile (i.e., a minimum jerk profile [35]) with the desired movement amplitude and duration. In contrast, optimal control generates the trajectories online based on optimal state estimation and optimal control gains [3, 4, 6]. Optimal control gains are derived from a cost function that may account for both accuracy and control effort. The estimated state is derived from the internal model and sensory feedback, according to optimal Kalman gains that account for the processs and measurement noise. Here we demonstrate that OFC with either CC or IC provide an alternative model for the correlations between dysmetria and early velocity reported in [23].

Our analysis focused on periodic IC, though HMC may be dominated by aperiodic IC triggered by crossing prediction error thresholds [14, 15, 22]. The analysis of periodic IC is justified since refractory mechanisms are assumed to set a lower limit on the sampling period [5, 13]. Thus, our analysis considers the limit of small thresholds, when the lower limit on the sampling period is reached consistently. Comparing event- and clock-driven IC with the same number of updates per movement, event driven IC is expected to be more robust to plant inaccuracies since it would provide more updates when the deviations are larger, though this would require further investigation.

The assumptions that the measurement and process noise are Gaussian white noise (rather than signal dependent noise) would affect the observer and feedback gains. Those gains are also affected by other assumptions that are commonly made in HMC including: (i) weights in the cost function are time-independent, or at least vary only at the desired time to reach the target, (ii) damping is time-independent, contrary to evidence suggesting that it changes during the movement [31], and (iii) feedback gains are determined to optimize a quadratic cost function rather than other functions, especially those that lead to higher robustness. The effects of the observer and feedback gains were considered by analyzing cases with different measurement noise, which affect the observer gains, and simulating cases with different cost-functions, which affect the feedback gain.

Given the highly uncertain environment in which human operate, robust control may provide a more viable framework for HMC than optimal control. Optimal control is usually not robust to plant variations, since it is tailored to specific parameters. Here, we focused on optimal control due to its prevalence in theories of HMC. Nevertheless, robust controllers, based on *H*_∞_, as suggested for postural control [36], or robust satisficing, as suggested for two-alternative forced choice tasks [37], may be more relevant for HMC.

In summary, our main contribution is in demonstrating that IC is a viable model of HMC even when considering the effects of model inaccuracies. In addition, our study demonstrates that OFC with either CC or periodic IC reproduces the reported correlation between dysmetria and early velocity with inaccurately modeled mass or damping.

## Supporting information

S1 File Control signals during IC and CC. Control signals during IC and CC of accurately and inaccurately modeled plants.
(PDF)

S2 File Proofs. Proof of Lemma 1 and Proof of Lemma 2.
(PDF)

S3 File Equivalent discrete-time systems with delays. (PDF)
