# Supplementary material for: Effects of model inaccuracies on reaching movements with intermittent control
Source: PLoS One. 2019 Oct 30;14(10):e0224265. doi: 10.1371/journal.pone.0224265 (PMC6821106; doi:10.1371/journal.pone.0224265)
Supplement: S1 File — Control signals during IC and CC of accurately and inaccurately modeled plants. (PDF) [file pone.0224265.s001.pdf]

Effects of model inaccuracies on reaching movements with intermittent control

Igor Gindin[1],Miri Benyamini[1] Miriam Zacksenhouse[1*]

1 Faculty of Mechanical Engineering, Technion Israel's Institute of Technology, Haifa 32000, Israel
*mermz@technion.ac.il

S1: Control signals during IC and CC

Summary of relevant equations from main text

The dynamics of the LTI plant is described by the system matrix $\bar{A}$ and control matrix $\bar{B}$ (Eq. (1)), which may differ from the system matrix A and control matrix B of the internal model (Eq. (2)):

$$\dot{x}(t) = \bar{A}x(t) + \bar{B}u(t) + w(t) \tag{1}$$

$$\dot{x}_{IM}(t) = Ax_{IM}(t) + Bu(t) \tag{2}$$

where $x \in R^n$ is the state of the plant, $u(t) \in R^m$ is the control signal, $w(t) \in R^n$ is the process noise, and $x_{IM} \in R^n$ is the state of the internal model.

The compound effect of process and measurement delay is accounted for by introducing measurement delay τ:

$$y(t) = Cx(t-\tau) + v(t-\tau) \tag{3}$$

where $y(t) \in R^q$ is the measurement and $v(t) \in R^q$ is the measurement noise.

Observer combines the internal model (Eq. (2)) and delayed measurement (Eq. (3)) to generate the estimated state $\hat{x}$ according to:

$$\dot{\hat{x}}(t-\tau) = A\hat{x}(t-\tau) + Bu(t-\tau) + L(t)(y(t) - C\hat{x}(t-\tau)) \tag{4}$$

Predictor predicts the current state, $x_p(t)$, given the estimated state, $\hat{x}(t-\tau)$, and the control signal $u(\sigma)$ for $\sigma \in [t-\tau, t)$, based on the internal model (Eq. (2)):

$$x_p(t) = e^{A\tau}\hat{x}(t-\tau) + \int_{t-\tau}^{t} e^{A(t-\sigma)}Bu(\sigma)d\sigma \tag{5}$$

LTI systems, i.e., LTI plants with time-invariant observer and controller gains, L and K, can be described by the overall state $x_{ov}(t-\tau) = [x(t-\tau)' \;\; \hat{x}(t-\tau)']'$. Combining Eq.s (1), (3) and (4) yields:

$$\dot{x}_{ov}(t-\tau) = A_o x_{ov}(t-\tau) + B_o u(t-\tau) + w_{ov}(t-\tau) \tag{6}$$

where A_o and B_o are defined in the main text, and $w_{ov}(t-\tau) = [w(t-\tau)' \;\; Lv(t-\tau)']'$ is the overall process noise.

Intermitent control performs predictions at discrete times t_m, which can be evenly spaced (periodic IC, $t_m = mh$, where h is the sampling period) or event driven (not considered in this work). At t_m, the predictor receives $\hat{x}(t_m - \tau)$ from the observer and

generates $x_p(t_m)$ according to Eq. (5). The latter provides the initial condition for the hold state, $x_h(t)$ that determines the control signal:

$$u(t) = -K(t)x_h(t) \tag{7}$$

Between samples, $x_h(t)$ evolves continuously according the feedback matrix $(A_F(t) = A - BK(t))$, defining the SMH:

$$\begin{cases} \dot{x}_h(t) = A_F(t)x_h(t),\ t \in [t_{m-1}, t_m) \\ x_h(t_m^+) = x_p(t_m),\ \forall m \in Z^+ \end{cases} \tag{8}$$

Control signal during IC of accurately modeled plant

The control signal generated by IC is proportional to the hold state, which evolves according to the system-matched hold (SMH, Eq (8)) and is reset every $t_m = mh$ to the predicted state. When the plant is accurately modeled, the SMH follows the state accurately, except for the effects of noise. Indeed, Figure A indicates that the state of the hold (dashed lines) follows the state of the plant (solid lines) during IC (with $h = 0.2$[sec]) of reaching movements with accurately modeled (delay-free) plant. Each state includes 4 variables: position (top left), velocity (bottom left), muscle force (f, top right) and the internal variable of the muscle model (g, bottom right). Zooming in on the time of the first reset $t_1 = h = 0.2$[sec], Figure B reveals that there are small changes in the state of the hold at that instance. Those changes can be attributed to the deviations between the hold and observer states due to the effects of noise. Thus, the control signal generated by IC is close to the control signal generated by CC, as can be seen in Figure C (top panel). Zooming in on the interval $(0.15, 0.45)$[sec] (Figure C, bottom panel) reveals that the reset of the hold state at $t_1 = h = 0.2$[sec] and $t_2 = 2h = 0.4$[sec] causes abrupt, proportionally small changes in the control signal.

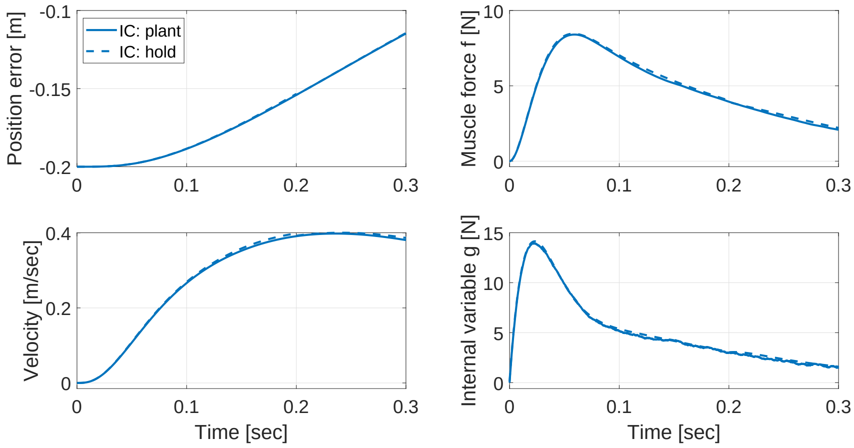

Figure A. The state of the plant (solid lines) and hold (dashed lines) during the initial 0.3[sec] of IC ($h = 0.2$[sec]) of accurately modeled delay-free plant with $m_{IM} = m_{PL} = 2$[Kg].
The state includes 4 variables: position (top left), velocity (bottom left), muscle force (f, top right) and the internal variable of the muscle model (g, bottom right). The hold state is reset at $t_1 = h = 0.2$[sec], but since the model is accurate the magnitude of the reset is negligible in this scale. See Figure B for a zoom in on the reset.

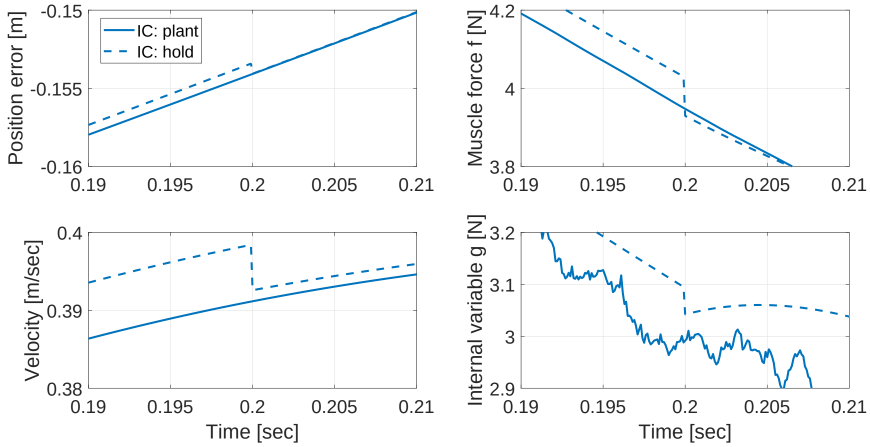

Figure B. Zoom in on the state of the plant (solid lines) and hold (dashed lines) around the first reset of the hold at $t_1 = h = 0.2$[sec] during IC ($h = 0.2$[sec]) of accurately modeled delay-free plant with $m_{IM} = m_{PL} = 2$[Kg].
See Figure A for the different variables of the state.

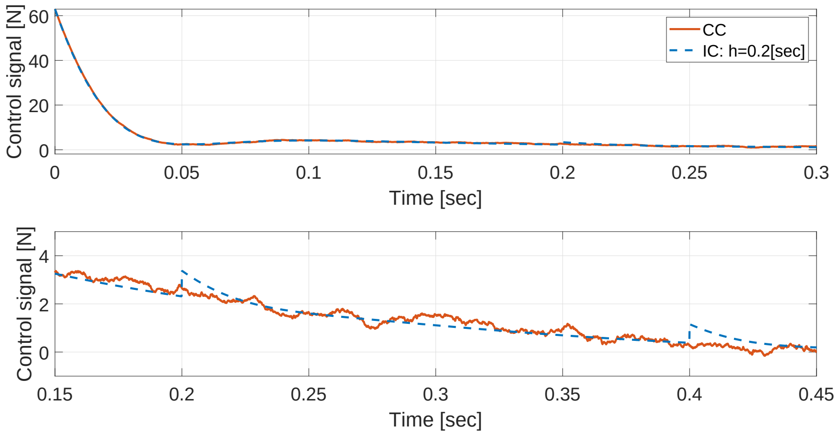

Figure C. Control signal during CC and IC ($h = 0.2$[sec]) of accurately modeled delay-free plant with $m_{IM} = m_{PL} = 2$[Kg].
Top: First 0.3[sec] of the movement. Bottom: The interval $(0.15, 0.45)$[sec] of the movement, during which the hold of IC is reset at $t_1 = h = 0.2$[sec] and $t_2 = 2h = 0.4$[sec].

Control signal during IC of inaccurately modeled plant

When the plant is modeled inaccurately, the SMH does not follow the state of the plant. Figure D demonstrates the deviations between the hold state (dashed lines) and the plant (solid lines) during the first 0.3[sec] of IC (with $h = 0.2$[sec]) of reaching movements with inaccurately modeled delay-free plant ($m_{IM} = 2$[Kg] and $m_{PL} = 1$[Kg]). The reset of the position and velocity of the hold state at $t_1 = h = 0.2$[sec] are apparent. Figure E focuses on the hold and plant states during the interval $(0.15, 0.45)$[sec] of the same movement, and demonstrates the reset at both $t_1 = h = 0.2$[sec] and $t_2 = 2h = 0.4$[sec]. The change in the muscle force (f, top right) is

more evident in this scale. Thus, the control signal generated by IC deviates from the control signal generated by CC, as is evident in Figure F and changes discontinuously when the hold state is reset. Note that the discontinuous change in the control signal at $t_1 = h = 0.2$[sec] is negative, while at $t_2 = 2h = 0.4$[sec] it is positive. This can be attributed to the opposite directions of the changes in the hold state at $t_1 = h = 0.2$[sec] versus $t_2 = 2h = 0.4$[sec].

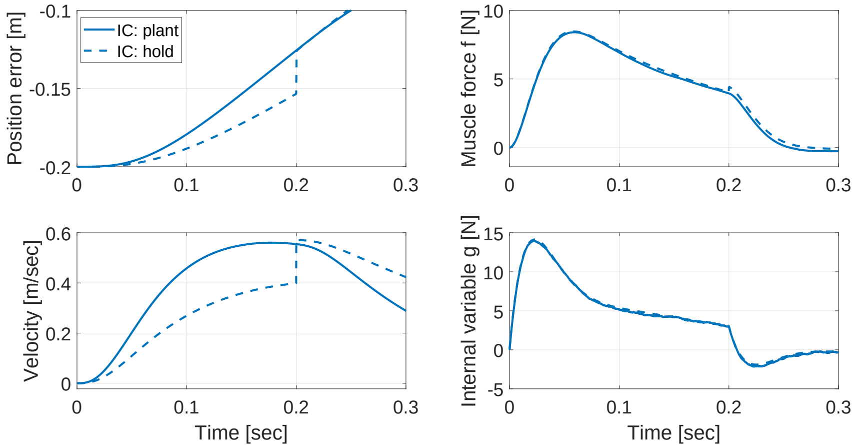

Figure D. The state of the plant (solid lines) and hold (dashed lines) during the initial 0.3[sec] of IC ($h = 0.2$[sec]) of inaccurately modeled delay-free plant with $m_{IM} = 2$[Kg] and $m_{PL} = 1$[Kg].
See Figure A for the different variables of the state. The hold state is reset at $t_1 = h = 0.2$[sec], as is evident in the discontinuous changes in position and velocity. See Figure E for a zoom in.

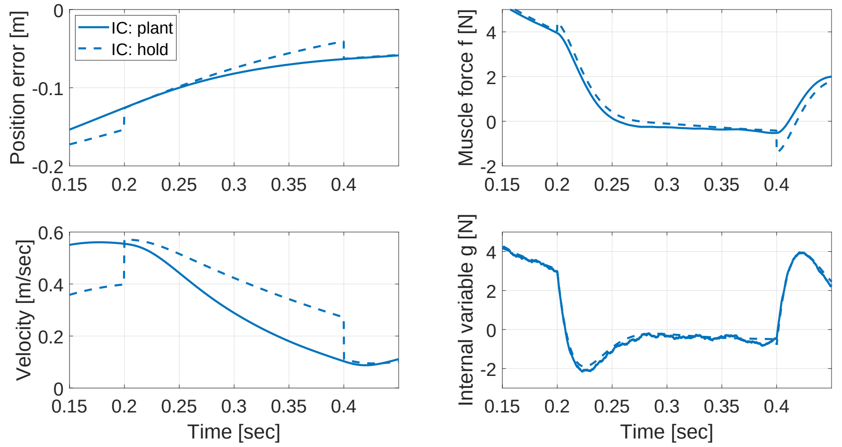

Figure E. Zoom in on the state of the plant (solid lines) and hold (dashed lines) around the interval (0.15, 0.45)[sec] during IC ($h = 0.2$[sec]) of inaccurately modeled delay-free plant with $m_{IM} = m_{PL} = 2$[Kg].
See Figure A for the different variables of the state. The hold state is reset at $t_1 = h = 0.2$[sec] and $t_2 = 2h = 0.4$[sec], as is evident in the discontinuous changes in position (top left), velocity (bottom left) and muscle force (top right).

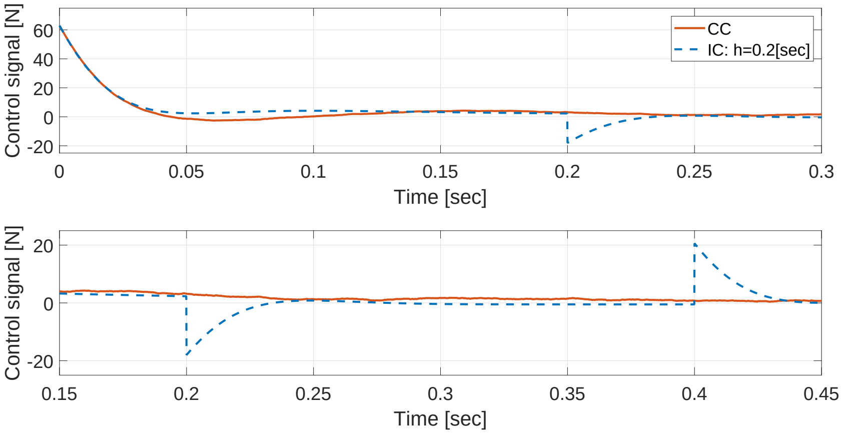

Figure F. Control signal during CC and IC ($h = 0.2$[sec]) of inaccurately modeled delay-free plant with $m_{IM} = m_{PL} = 2$[Kg].
Top: First 0.3[sec] of the movement. Bottom: The interval $(0.15, 0.45)$[sec] of the movement, during which the hold of IC is reset at $t_1 = 0.2$[sec] and $t_2 = 0.4$[sec].
