# Supplementary material for: Effects of model inaccuracies on reaching movements with intermittent control
Source: PLoS One. 2019 Oct 30;14(10):e0224265. doi: 10.1371/journal.pone.0224265 (PMC6821106; doi:10.1371/journal.pone.0224265)
Supplement: S2 File — Proof of Lemma 1 and Proof of Lemma 2. (PDF) [file pone.0224265.s002.pdf]

Effects of model inaccuracies on reaching movements with intermittent control

Igor Gindin[1],Miri Benyamini[1] Miriam Zacksenhouse[1*]

1 Faculty of Mechanical Engineering, Technion Israel's Institute of Technology, Haifa 32000, Israel
*mermz@technion.ac.il

S2: Proofs

Summary of relevant equations from main text

The dynamics of the LTI plant is described by the system matrix $\bar{A}$ and control matrix $\bar{B}$ (Eq. (1)), which may differ from the system matrix A and control matrix B of the internal model (Eq. (2)):

$$\dot{x}(t) = \bar{A}x(t) + \bar{B}u(t) + w(t) \tag{1}$$

$$\dot{x}_{IM}(t) = Ax_{IM}(t) + Bu(t) \tag{2}$$

where $x \in R^n$ is the state of the plant, $u(t) \in R^m$ is the control signal, $w(t) \in R^n$ is the process noise, and $x_{IM} \in R^n$ is the state of the internal model.

The compound effect of process and measurement delay is accounted for by introducing measurement delay τ:

$$y(t) = Cx(t-\tau) + v(t-\tau) \tag{3}$$

where $y(t) \in R^q$ is the measurement and $v(t) \in R^q$ is the measurement noise.

Observer combines the internal model (Eq. (2)) and delayed measurement (Eq. (3)) to generate the estimated state $\hat{x}$ according to:

$$\dot{\hat{x}}(t-\tau) = A\hat{x}(t-\tau) + Bu(t-\tau) + L(t)(y(t) - C\hat{x}(t-\tau)) \tag{4}$$

Predictor predicts the current state, $x_p(t)$, given the estimated state, $\hat{x}(t-\tau)$, and the control signal $u(\sigma)$ for $\sigma \in [t-\tau, t)$, based on the internal model (Eq. (2)):

$$x_p(t) = e^{A\tau}\hat{x}(t-\tau) + \int_{t-\tau}^{t} e^{A(t-\sigma)}Bu(\sigma)d\sigma \tag{5}$$

LTI systems, i.e., LTI plants with time-invariant observer and controller gains, L and K, can be described by the overall state $x_{ov}(t-\tau) = [x(t-\tau)' \;\; \hat{x}(t-\tau)']'$. Combining Eq.s (1), (3) and (4) yields:

$$\dot{x}_{ov}(t-\tau) = A_o x_{ov}(t-\tau) + B_o u(t-\tau) + w_{ov}(t-\tau) \tag{6}$$

where A_o and B_o are defined in the main text, and $w_{ov}(t-\tau) = [w(t-\tau)' \;\; Lv(t-\tau)']'$ is the overall process noise.

Intermitent control performs predictions at discrete times t_m, which can be evenly spaced (periodic IC, $t_m = mh$, where h is the sampling period) or event driven (not considered in this work). At t_m, the predictor receives $\hat{x}(t_m - \tau)$ from the observer and

generates $x_p(t_m)$ according to Eq. (5). The latter provides the initial condition for the hold state, $x_h(t)$ that determines the control signal:

$$u(t) = -K(t)x_h(t) \tag{7}$$

Between samples, $x_h(t)$ evolves continuously according the feedback matrix $(A_F(t) = A - BK(t))$, defining the SMH:

$$\begin{cases} \dot{x}_h(t) = A_F(t)x_h(t), \ t \in [t_{m-1}, t_m) \\ x_h(t_m^+) = x_p(t_m), \ \forall m \in Z^+ \end{cases} \tag{8}$$

Proof of Lemma 1: The proof is based on solving the dynamics of $x_{ov}(t) = [x(t)' \ \hat{x}(t)']'$ in continuous time, discretizing the solution at the period h of the periodic IC, and using system-matched hold (SMH, Eq. (8)) to determine the control signal between samples (Eq. (7)).

In the delay-free case, the solution of Eq. (6) is:

$$x_{ov}(t) = e^{A_o t}x_{ov}(0) + \int_0^t e^{A_o(t-\sigma)}B_o u(\sigma)d\sigma + \int_0^t e^{A_o(t-\sigma)}w_{ov}(\sigma)d\sigma.$$

Sampling x_{ov} at a fixed period h the mth sample of $x_{ov}(t)$, denoted $x_{ov}[m] = x_{ov}(mh)$, is given by:

$$x_{ov}[m] = e^{A_o mh}x_{ov}(0) + \int_0^{mh} e^{A_o(mh-\sigma)}B_o u(\sigma)d\sigma + \int_0^{mh} e^{A_o(mh-\sigma)}w_{ov}(\sigma)d\sigma.$$

The next sample, $x_{ov}[m+1]$, can be related to $x_{ov}[m]$ by:

$$x_{ov}[m+1] = e^{A_o h}x_{ov}[m] + \int_{mh}^{mh+h} e^{A_o(mh+h-\sigma)}B_o u(\sigma)d\sigma + \int_{mh}^{mh+h} e^{A_o(mh+h-\sigma)}w_{ov}(\sigma)d\sigma. \tag{9}$$

The last term is the noise term in the $m+1$ step, denoted by $w_{ov}[m]$, which is independent of the overall state. In periodic IC, the control signal $u(t)$ $t \in (mh, mh+h)$ depends on $x_h(t)$ (Eq. (7)), which evolves according to the SMH (Eq. (8)) from the last sample of the estimated state $\hat{x}(t_m) = \hat{x}(mh) \equiv \hat{x}[m]$. Solving (Eq. (8)) for $x_h(t)$, the control signal $u(t)$ is derived and inserted in Eq. (9) to get:

$$x_{ov}[m+1] = e^{A_o h}x_{ov}[m] - \left(\int_{mh}^{mh+h} e^{A_o(mh+h-\sigma)}B_o K e^{A_F(\sigma-mh)}d\sigma\right)\hat{x}[m] + w_{ov}[m].$$

Applying the change of variables $\theta = \sigma - mh$, this can be expressed as:

$$x_{ov}[m+1] = e^{A_o h}\left(I_{2n} - \int_0^h e^{-A_o\theta}B_o K e^{A_F\theta}d\theta[0 \ I_n]\right)x_{ov}[m] + w_{ov}[m]. \tag{10}$$

The expression in parentheses in Eq. (10) is a fixed matrix, so Eq. (10) is an autonomous discrete LTI system. The integral expression in the parentheses can be solved using exponential of triangular matrices [1], but here we solve it using Sylvester equation. Using integration by parts and assuming that A_F is non-singular, the integral is expressed as:

$$\begin{aligned} S = \int_0^h \underbrace{e^{-A_o\theta}B_o K}_{f}\underbrace{e^{A_F\theta}}_{g'}d\theta = \underbrace{e^{-A_o\theta}B_o K}_{f}\underbrace{e^{A_F\theta}A_F{}^{-1}}_{g}\Big|_0^h - \\ \int_0^h \underbrace{-A_o e^{-A_o\theta}B_o K}_{f'}\underbrace{e^{A_F\theta}A_F{}^{-1}}_{g}d\theta \end{aligned} \tag{11}$$

where

$$g = \int e^{A_F\theta} d\theta = e^{A_F\theta} {A_F}^{-1}.$$

The second term in Eq. (11) is $-A_o S {A_F}^{-1}$, so:

$$S = (e^{-A_o h} B_o K e^{A_F h} - B_o K){A_F}^{-1} + A_o S {A_F}^{-1}.$$

Multiplying both sides by A_F (from the right) and moving all terms to one side yields:

$$A_o S + S(-A_F) + (e^{-A_o h} B_o K e^{A_F h} - B_o K) = 0, \tag{12}$$

which is a sylvester equation $\gamma S + S\beta + \alpha = 0$ with:

$$\alpha = e^{-A_o h} B_o K e^{A_F h} - B_o K, \quad \beta = -A_F, \ \gamma = A_o.$$

Given S (obtained by Matlab command *lyap* or *sylvester*), Eq. (10) reduces to $x_{ov}[m+1] = A_p x_{ov}[m] + w_{ov}[m]$, where

$$A_p = e^{A_c h}(I_{2n} - S[0 \ I_n]). \tag{13}$$

□

Proof of Lemma 2: In periodic IC, predictions are performed at evenly-spaced discrete times $t_m \equiv mh$, where h is the samplng period. At t_m the predictor receives $\hat{x}[m] \equiv \hat{x}(t_m - \tau)$, where τ is the delay, and predicts the state at t_m, $x_p[m]$, according to Eq. (5):

$$x_p[m] = e^{A\tau}\hat{x}[m] + \int_{mh-\tau}^{mh} e^{A(mh-\sigma)} B u(\sigma) d\sigma. \tag{14}$$

The control signal is determined from the hold state: $u(\sigma) = -K x_h(\sigma)$ (Eq. (7)). Assuming $\tau < h$, $x_h(\sigma)$ in the interval $\sigma \in [mh - \tau, mh)$ evolves from the previous prediction $x_p[m-1]$ according to Eq. (8), so $x_h(\sigma) = e^{A_F(\sigma-(m-1)h)} x_p[m-1]$. Thus:

$$x_p[m] = e^{A\tau}\hat{x}[m] - \int_{mh-\tau}^{mh} e^{A(mh-\sigma)} B K e^{A_F(\sigma-(m-1)h)} x_p[m-1] d\sigma.$$

Applying the change of variables $\theta = \sigma - mh$, this can be expressed as:

$$x_p[m] = e^{A\tau}\hat{x}[m] - \left(\int_{-\tau}^{0} e^{-A\theta} B K e^{A_F\theta} d\theta \right) e^{A_F h} x_p[m-1]. \tag{15}$$

The integral expression in the parentheses, denoted by S_1, can be solved using Sylvester equation. Following the development in the proof of Lemma 1 (Eq. (11)), S_1 solves the sylvester equation $\gamma_1 S_1 + S_1 \beta_1 + \alpha_1 = 0$ with:

$$\alpha_1 = BK - e^{A\tau} B K e^{-A_F\tau}, \quad \beta_1 = -A_F, \ \gamma_1 = A,$$

Given S_1, Eq. (15) can be expressed as:

$$x_p[m] = e^{A\tau}\hat{x}[m] - S_1 e^{A_F h} x_p[m-1]. \tag{16}$$

The second part of the proof develops a difference equation for $\hat{x}[m]$ as part of a difference equation for $x_{ov}[m] \equiv [x(t_m - \tau)' \ \hat{x}(t_m - \tau)']'$. Solving Eq. (6) from the intial condition $x_{ov}[m]$ and evaluating the solution at $t = t_{m+1} - \tau$, the next sample

$x_{ov}[m+1]$ is (the derivation is simillar to that of Eq. (9) in the proof of Lemma 1, except for the delay):

$$x_{ov}[m+1] = e^{A_o h} x_{ov}[m] + \int_{mh-\tau}^{mh+h-\tau} e^{A_o(mh+h-\tau-\sigma)} B_o u(\sigma) d\sigma + w_{ov}[m] \tag{17}$$

where $w_{ov}[m] \equiv \int_{mh-\tau}^{mh+h-\tau} e^{A_o(mh+h-\tau-\sigma)} w_{ov}(\sigma) d\sigma$ is the noise term.

Given that $\tau < h$, $x_h(\sigma)$ in the interval $\sigma \in [mh-\tau, mh]$ evolves from the initial state $x_p[m-1]$ according to the elapsed time $\sigma - (m-1)h$, while $x_h(\sigma)$ in the interval $\sigma \in [mh, mh+h-\tau]$ evolves from the initial state $x_p[m]$ according to the elapsed time $\sigma - mh$. Solving Eq. (8) for $x_h(\sigma)$ in those two intervals, the control signal is $u(\sigma) = -K x_h(\sigma)$ (Eq. (7)). Thus, the integral in Eq. (17) is:

$$\begin{aligned} &-\left(\int_{mh-\tau}^{mh} e^{A_o(mh+h-\tau-\sigma)} B_o K e^{A_F(\sigma-(m-1)h)} d\sigma\right) x_p[m-1] - \\ &\left(\int_{mh}^{mh+h-\tau} e^{A_o(mh+h-\tau-\sigma)} B_o K e^{A_F(\sigma-mh)} d\sigma\right) x_p[m]. \end{aligned} \tag{18}$$

Applying the change of variables $\theta = \sigma - (mh-\tau)$ this can be expressed as:

$$-e^{A_o h}\left[\left(\int_0^{\tau} e^{-A_o\theta} B_o c K e^{A_F\theta} d\theta\right) e^{A_F(h-\tau)} x_p[m-1] + \left(\int_{\tau}^{h} e^{-A_o\theta} B_o K e^{A_F\theta} d\theta\right) e^{-A_F\tau} x_p[m]\right] \tag{19}$$

The integral expressions in the first and second parenthesis, denoted by S_2 and S_3, can be solved using sylveter equations $\gamma_i S_i + S_i \beta_i + \alpha_i = 0$, $i = 2, 3$ with:

$$\alpha_2 = e^{-A_o\tau} B_o K e^{A_F\tau} - B_o K, \quad \beta_2 = -A_F,\ \gamma_2 = A_o,$$

$$\alpha_3 = e^{-A_o h} B_o K e^{A_F h} - e^{-A_o\tau} B_o K e^{A_F\tau}, \quad \beta_3 = -A_F,\ \gamma_3 = A_o.$$

Given S_2 and S_3, Eq. (19) can be expressed as:

$$-e^{A_o h}\left[S_2 e^{A_F(h-\tau)} x_p[m-1] + S_3 e^{-A_F\tau} x_p[m]\right] \tag{20}$$

Inserting Eq. (20) in Eq. (17) yields:

$$x_{ov}[m+1] = e^{A_o h}\left(x_{ov}[m] - S_2 e^{A_F(h-\tau)} x_p[m-1] - S_3 e^{-A_F\tau} x_p[m]\right) + w_{ov}[m]. \tag{21}$$

Defining $x_{tot}[m] = [x_{ov}[m]'\ x_p[m-1]']'$, Eq. (16) and Eq. (21) can be combined to derive the difference equation defining the dynamics of the state-observer-predictor system: $x_{tot}[m+1] = A_{tot} x_{tot}[m] + w_{tot}[m]$, where

$$A_{tot} = \begin{pmatrix} e^{A_o h}(I_{2n} - S_3 e^{-A_F\tau} e^{A\tau}[0_n\ I_n]) & e^{A_o h}(-S_2 e^{-A_F\tau} + S_3 e^{-A_F\tau} S_1) e^{A_F h} \\ e^{A\tau}[0_n\ I_n] & -S_1 e^{A_F h} \end{pmatrix}, \tag{22}$$

and $w_{tot} = [w_{ov}; 0_{n\times 1}]$. $\square$

References

1. Van Loan C, Computing integrals involving the matrix exponential, IEEE transactions on automatic control, 23(3), 395-404 (1978).
