# Supplementary material for: Effects of model inaccuracies on reaching movements with intermittent control
Source: PLoS One. 2019 Oct 30;14(10):e0224265. doi: 10.1371/journal.pone.0224265 (PMC6821106; doi:10.1371/journal.pone.0224265)
Supplement: S3 File — (PDF) [file pone.0224265.s003.pdf]

Effects of model inaccuracies on reaching movements with intermittent control

Igor Gindin[1],Miri Benyamini[1] Miriam Zacksenhouse[1*]

1 Faculty of Mechanical Engineering, Technion Israel's Institute of Technology, Haifa 32000, Israel
*mermz@technion.ac.il

S3: Equivalent discrete-time systems with delays

Summary of relevant equations from main text

The dynamics of the LTI plant is described by the system matrix $\bar{A}$ and control matrix $\bar{B}$ (Eq. (1)), which may differ from the system matrix A and control matrix B of the internal model (Eq. (2)):

$$\dot{x}(t) = \bar{A}x(t) + \bar{B}u(t) + w(t) \tag{1}$$

$$\dot{x}_{IM}(t) = Ax_{IM}(t) + Bu(t) \tag{2}$$

where $x \in R^n$ is the state of the plant, $u(t) \in R^m$ is the control signal, $w(t) \in R^n$ is the process noise, and $x_{IM} \in R^n$ is the state of the internal model.

The compound effect of process and measurement delay is accounted for by introducing measurement delay τ:

$$y(t) = Cx(t-\tau) + v(t-\tau) \tag{3}$$

where $y(t) \in R^q$ is the measurement and $v(t) \in R^q$ is the measurement noise.

Observer combines the internal model (Eq. (2)) and delayed measurement (Eq. (3)) to generate the estimated state $\hat{x}$ according to:

$$\dot{\hat{x}}(t-\tau) = A\hat{x}(t-\tau) + Bu(t-\tau) + L(t)(y(t) - C\hat{x}(t-\tau)) \tag{4}$$

Predictor predicts the current state, $x_p(t)$, given the estimated state, $\hat{x}(t-\tau)$, and the control signal $u(\sigma)$ for $\sigma \in [t-\tau, t)$, based on the internal model (Eq. (2)):

$$x_p(t) = e^{A\tau}\hat{x}(t-\tau) + \int_{t-\tau}^{t} e^{A(t-\sigma)}Bu(\sigma)d\sigma \tag{5}$$

LTI systems, i.e., LTI plants with time-invariant observer and controller gains, L and K, can be described by the overall state $x_{ov}(t-\tau) = [x(t-\tau)' \;\; \hat{x}(t-\tau)']'$. Combining Eq.s (1), (3) and (4) yields:

$$\dot{x}_{ov}(t-\tau) = A_o x_{ov}(t-\tau) + B_o u(t-\tau) + w_{ov}(t-\tau) \tag{6}$$

where A_o and B_o are defined in the main text, and $w_{ov}(t-\tau) = [w(t-\tau)' \;\; Lv(t-\tau)']'$ is the overall process noise.

Intermitent control performs predictions at discrete times t_m, which can be evenly spaced (periodic IC, $t_m = mh$, where h is the sampling period) or event driven (not considered in this work). At t_m, the predictor receives $\hat{x}(t_m - \tau)$ from the observer and

generates $x_p(t_m)$ according to Eq. (5). The latter provides the initial condition for the hold state, $x_h(t)$ that determines the control signal:

$$u(t) = -K(t)x_h(t) \tag{7}$$

Between samples, $x_h(t)$ evolves continuously according the feedback matrix ($A_F(t) = A - BK(t)$), defining the SMH:

$$\begin{cases} \dot{x}_h(t) = A_F(t)x_h(t), \ t \in [t_{m-1}, t_m) \\ x_h(t_m^+) = x_p(t_m), \ \forall m \in Z^+ \end{cases} \tag{8}$$

Equivalent discrete-time systems with delays

To facilitate the stability analysis of continuous time-delayed systems, they are converted to equivalent discrete-time systems using the standard zero-order hold [1]. Given the delay τ, the discretization time Δ is selected so $k_\tau = \tau/\Delta$ is an integer number. Thus, the equivalent discrete-time system of the plant, (1) and (3), and internal model, (2), is described by the following difference equations:

$$x(k+1) = \bar{A}_d x(k) + \bar{B}u(k) + w_d(k) \tag{9}$$

$$y(k) = Cx(k - k_\tau) + v_d(k - k_\tau) \tag{10}$$

$$x_{IM}(k+1) = A_d x_{IM}(k) + B_d u(k) \tag{11}$$

where $\bar{A}_d = exp(\bar{A}\Delta)$, $\bar{B}_d = \bar{A}^{-1}(exp(\bar{A}\Delta) - I)\bar{B}$, $A_d = exp(A\Delta)$ and $B_d = A^{-1}(exp(A\Delta) - I)B$. The covariance matrices of the discrete process and measurement noise are $W_d = W\Delta$ and $V_d = V/\Delta$, respectively [1].

Stability is analyzed for time-invariant systems with constant observer and controller gain matrices, L_d and K_d, respectively. These gain matrices can be computed using standard optimal estimation and control tools under the assumption of accurate model for an infinite horizon cost function. In this case, the optimal Kalman gain matrix is $L_d = P_d C'(CP_dC' + V_d)^{-1}$ where P_d is the solution of the discrete time algebraic Riccati equation: $P_d = A_d P_d A_d' - (A_d P_d C')(CP_dC' + V_d)^{-1}(CP_dA_d' + W_d)$, while the optimal feedback gain matrix is $K_d = (B_d' S_d B_d + R)^{-1} B' S_d A_d$ where S_d is the solution of the discrete time algebraic Riccati equation: $S_d = A_d' S_d A_d - (A_d' S_d B_d)(B_d' S_d B_d + R_\infty)^{-1}(B_d' S_d A_d + Q_\infty)$.

Due to the measurement delay, the current measurement $y(k)$ depends on the delayed state $x(k - k_\tau)$. Hence the observer updates the estimated state $\hat{x}(k - k_\tau)$ according to:

$$\hat{x}(k - k_\tau + 1) = A_d\hat{x}(k - k_\tau) + B_d u(k - k_\tau) + L_d(y(k) - C\hat{x}(k - k_\tau)). \tag{12}$$

The predicted state is the solution of (11), given the estimated state $\hat{x}(k - k_\tau)$:

$$x_p(k) = A_d^{k_\tau}\hat{x}(k - k_\tau) + \sum_{i=0}^{k_\tau - 1} A_d^{k_\tau - i - 1} B_d u(i + k - k_\tau). \tag{13}$$

The control signal is proportional to the predicted state:

$$u(k) = -K_d x_p(k). \tag{14}$$

Equations (13) and (14) imply that $x_p(k)$ depends on $x_p(k - k_\tau), ..., x_p(k-1)$. This dependence is captured by defining the extended state $x_{exv}(k) = [x(k - k_\tau)' \ \hat{x}(k - k_\tau)' \ x_p(k-1)' \ \ldots \ x_p(k - k_\tau)']'$. Thus, the dynamics of the overall discrete system described by (9) - (14) can be expressed as

$x_{ex}(k+1) = A_{ex}x_{ex}(k) + w_{ex}(k)$, where $w_{ex}(k) = [w_d(k-k_\tau)' \; L_d v_d(k-k_\tau)' \; 0 \ldots \; 0]$ is the overall discrete process noise and

$$A_{ex} = \begin{pmatrix} \bar{A}_d & 0 & 0 & 0 & \ldots & 0 & -\bar{B}_d K_d \\ L_d C & (A_d - L_d C) & 0 & 0 & \ldots & 0 & -B_d K_d \\ 0 & A_d^{k_\tau} & -B_d K_d & -A_d B_d K_d & \ldots & -A_d^{k_\tau - 2} B_d K_d & -A_d^{k_\tau - 1} B_d K_d \\ 0 & 0 & I & 0 & \ldots & 0 & 0 \\ 0 & 0 & 0 & I & \ldots & 0 & 0 \\ \vdots & \vdots & \vdots & \vdots & \vdots & \vdots & \vdots \\ 0 & 0 & 0 & 0 & \ldots & I & 0 \end{pmatrix} \tag{15}$$

References

1. Stengel R F, Optimal Control and Estimation. Courier Corporation (1994).
